# Structure Prediction and Protein Engineering Yield New Insights into Microcin J25 Precursor Recognition

**DOI:** 10.1021/acschembio.4c00251

**Published:** 2024-08-20

**Authors:** Hui-Ni Tan, Wei-Qi Liu, Josh Ho, Yi-Ju Chen, Fang-Jie Shieh, Hsiao-Tzu Liao, Shu-Ping Wang, Julian D. Hegemann, Chin-Yuan Chang, John Chu

**Affiliations:** † Department of Chemistry, National Taiwan University, Taipei 10617, Taiwan; ‡ Department of Biological Science and Technology, National Yang Ming Chiao Tung University, Hsinchu 300193, Taiwan; § Institute of Biomedical Sciences, Academia Sinica, Taipei 115201, Taiwan; ∥ Helmholtz Institute for Pharmaceutical Research Saarland, Helmholtz Centre for Infection Research, Saarland University Campus, 66123 Saarbrücken, Germany

## Abstract

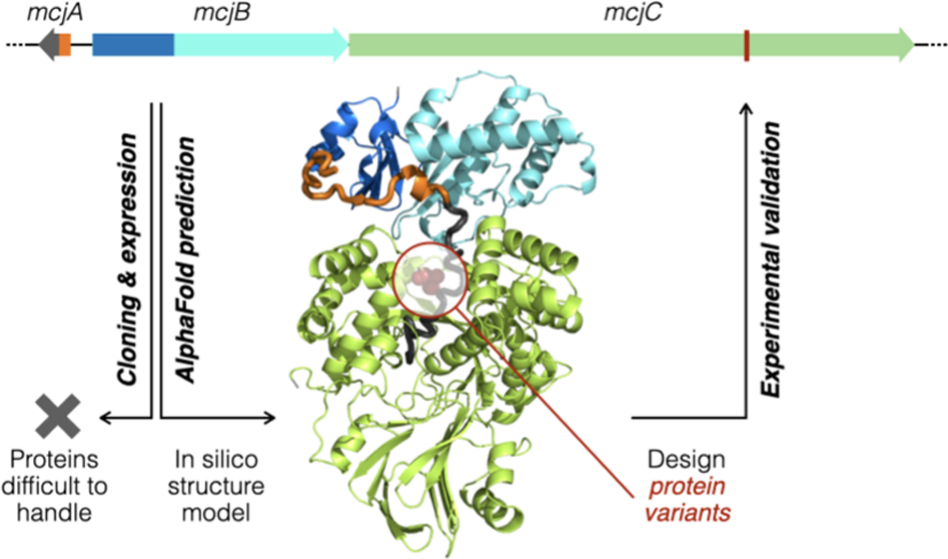

Microcin J25 (MccJ25), a lasso peptide antibiotic with a unique structure that resembles the lariat knot, has been a topic of intense interest since its discovery in 1992. The precursor (McjA) contains a leader and a core segment. McjB is a protease activated upon binding to the leader, and McjC converts the core segment into the mature MccJ25. Previous studies suggested that these biosynthetic steps likely proceed in a (nearly) concerted fashion; however, there is only limited information regarding the structural and molecular intricacies of MccJ25 biosynthesis. To close this knowledge gap, we used AlphaFold2 to predict the structure of the precursor (McjA) in complex with its biosynthetic enzymes (McjB and McjC) and queried the critical predicted features by protein engineering. Based on the predicted structure, we designed protein variants to show that McjB can still be functional and form a proficient biosynthetic complex with McjC when its recognition and protease domains were circularly permutated or split into separate proteins. Specific residues important for McjA recognition were also identified, which permitted us to pinpoint a compensatory mutation (McjB_M108T_) to restore McjA/McjB interaction that rescued an otherwise nearly nonproductive precursor variant (McjA_T–2M_). Studies of McjA, McjB, and McjC have long been mired by them being extremely difficult to handle experimentally, and our results suggest that the AF2 predicted ternary complex structure may serve as a reasonable starting point for understanding MccJ25 biosynthesis. The prediction-validation workflow presented herein combined artificial intelligence and laboratory experiments constructively to gain new insights.

## Introduction

**Figure 1:**
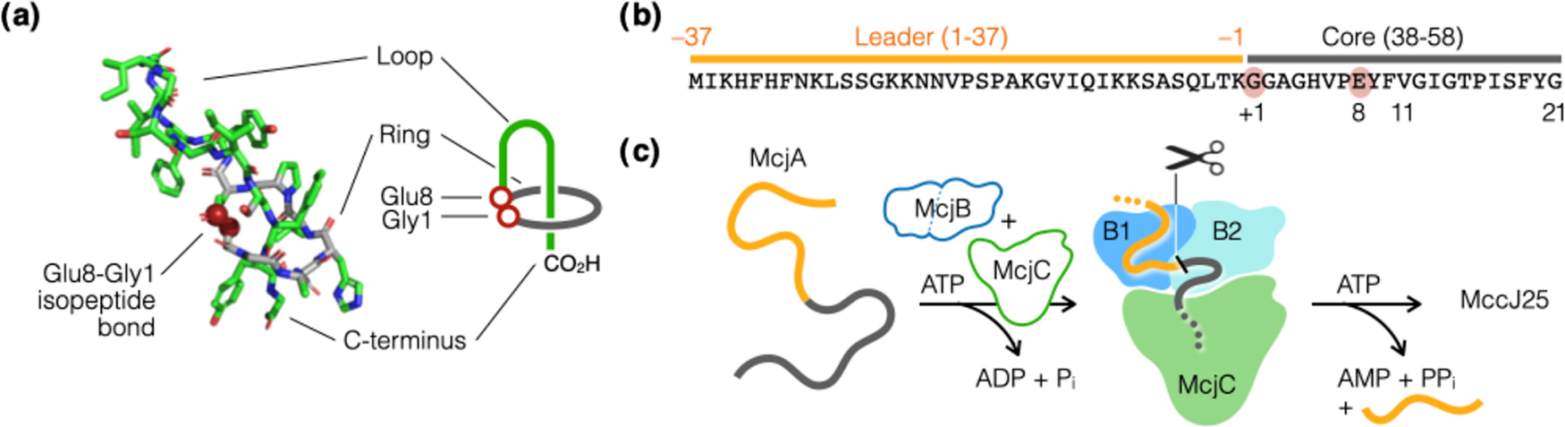
Unique structure and biosynthetic pathway. (a) MccJ25 contains a macrolactam (the ring) that results from the formation of an isopeptide bond between its Gly1 N-terminal amine and Glu8 side-chain carboxylate. The rest of the peptide folds into a hairpin-like structure (the loop) and passes through the macrolactam to form a “threaded lasso” configuration (PDB: 1PP5).^1^ (b) The MccJ25 precursor (McjA) is a 58-mer peptide, wherein the leader (orange) and the core (black) peptides are numbered −37 to −1 and +1 to 21, respectively. (c) MccJ25 maturation is carried out by two enzymes: (1) McjB contains two domains, B1 (blue) and B2 (cyan), and are responsible for leader recognition and cleavage, respectively, and (2) McjC (green) catalyzes the formation of the isopeptide bond. Note that McjA, McjB, and McjC are color-coded the same way throughout this manuscript.

Microcin J25 (MccJ25) is a potent antibiotic with a lasso structure (Figure 1a).^1−3^ It is a peptide natural product that consists of 21 amino acids, whose C-terminal tail passes through a macrocycle that results from the formation of an isopeptide bond between the Gly1 N-terminal amine and the Glu8 side-chain carboxylate. This threaded lasso configuration is stabilized by two bulky residues immediately above (Phe19) and below (Tyr20) the macrolactam ring and remains intact against chemical and thermal denaturation.^4,5^ MccJ25 was the first antibiotic known to inhibit transcription by blocking the secondary channel of the bacterial RNA polymerase complex.^6^ As a natural product with a unique structure and mechanism of action, MccJ25 has fascinated scientists since its discovery in 1992.^7,8^ However, despite extensive interest over the past three decades, the structural and molecular details of MccJ25 biosynthesis have remained elusive. Herein, we combined AlphaFold2 (AF2) structure prediction^9^ and protein engineering, including domain swapping, cyclic permutation, site-directed mutagenesis, and synthetic rescue, to gain new insights into the interaction between the MccJ25 precursor peptide (McjA) and its biosynthetic enzymes (McjB and McjC).

MccJ25 is a ribosomally synthesized and post-translationally modified peptide (RiPP). A lasso peptide biosynthetic gene cluster (BGC) in general encodes three genes (A–C).^10,11^ They are named *mcjA*, *mcjB*, and *mcjC* in the MccJ25 BGC,^12^ which contains an additional membrane transporter (McjD) that confers self-resistance by exporting the mature MccJ25.^13^ McjA is the precursor peptide (Figure 1b), which includes a leader segment (McjA_(−37)to(−1)_) and a core segment (McjA_1–21_). Residues of the core peptide were numbered 1 to 21 and those of the leader peptide were assigned negative numbers. MccJ25 maturation is catalyzed by the biosynthetic enzymes McjB and McjC (Figure 1c).^14,15^ Specifically, McjB cleaves the amide bond that connects the leader and the core, and McjC catalyzes the formation of the macrolactam that entraps its own tail into a threaded configuration. Furthermore, in vitro assays showed that McjB and McjC are only active in the presence of each other,^15^ suggesting that they likely act in a (nearly) concerted fashion to catalyze the maturation of MccJ25. While this phenomenon is interesting from a molecular mechanism viewpoint, it makes elucidating the structural and molecular details of MccJ25 biosynthesis significantly more challenging.

The conversion of a RiPP precursor into the final natural product generally entails the following steps.^16^ The leader peptide first interacts with a conserved domain in one of the biosynthetic enzymes, called the RiPP precursor recognition element (RRE).^17^ This interaction is the gatekeeping event that triggers further post-translational modification(s). In lasso peptide biosynthesis, a protease is activated upon leader recognition by the RRE to cleave the precursor into the leader and the core peptides.^18^ Interestingly, the RRE and protease domains may exist either as a single didomain protein, such as McjB in the MccJ25 BGC, or discretely as separate proteins, such as PadeB1 and PadeB2 in the paeninodin BGC.^19^ The open reading frame (ORF) that encodes a fused enzyme is termed the “B” gene, whereas separate RRE and protease are usually termed the B1 and B2 proteins, respectively, a naming convention we adhered to throughout this manuscript. Note that the RRE and protease in lasso peptide BGC are occasionally termed the B and E proteins.

In vitro assays for leader recognition and/or cleavage, either by separate B1/B2 proteins or a fused B enzyme, have been developed to study the biosynthesis of fusilassin (also known as fuscanodin), lariatin, paeninodin, burhizin, and therbactin.^20−26^ These assays revealed residues key to leader recognition by the RRE to activate the protease. Furthermore, X-ray crystal structures of B1 proteins, including RRE/leader peptide cocrystal structures for fusilassin and therbactin biosynthesis, were reported recently and provided details at atomic resolution (Table S1).^18,27^ Unfortunately, as the lasso peptide that initiated the field of supramolecular natural product research, not nearly as much is known about MccJ25 biosynthesis. Herein, AF2 was used to predict the structure of the McjA/McjB/McjC ternary complex, and its critical features were confirmed by MccJ25 production using engineered biosynthetic enzymes designed based on the predicted structure. The AF2 prediction is therefore a reasonable model and can serve as the starting point toward understanding the structural basis of MccJ25 biosynthesis.

The study of MccJ25 biosynthesis has been mired by McjA, McjB, and McjC being very difficult to handle experimentally. McjA alone is unstructured and highly susceptible to proteases; most His-tagged McjB and McjC end up in inclusion bodies, supplying as little as tens of micrograms of pure proteins per liter of culture.^14^ These enzymes were obtained at low yields even when fused to a maltose binding protein (MBP),^15^ and MBP-McjC still forms high-molecular-weight aggregates consisting of 10 or more monomers (Figure S1). More challenging still, the enzymatic actions of McjB (leader activation and cleavage) and McjC (macrolactam formation) cannot be studied in separate assays as they depend upon each other for activation,^14,15^ hinting at (nearly) concerted biosynthetic steps. As such, it may require a high-resolution structure of the McjA/McjB/McjC ternary complex, as opposed to individual proteins, to fully understand the molecular details of MccJ25 maturation, making an already formidable task even more demanding.

## Results

### AF2 Predicts a McjA/McjB/McjC Ternary Complex

In light of these struggles, we used AF2 to foray into the structural and molecular details of MccJ25 biosynthesis.^9^ We are aware of a number of caveats regarding such an endeavor.^28^ An AF2 predicted structure is usually accurate when the subject is homologous to proteins whose structures have already been determined. Structure predictions of proteins with no experimentally characterized homologues, as well as protein–protein interaction, are generally taken with a grain of salt. In our case, AF2 was not only tasked with predicting the structure of the McjA/McjB/McjC ternary complex, but there were reasons to believe that each of the three components poses challenges. As a 58-residue linear peptide, McjA is intrinsically disordered and without a defined structure.^29^ Even though McjB is known to be a cysteine protease, it is an atypical member of this enzyme family as it requires ATP to operate and is inactive without the presence of McjC.^15^ As for McjC, it is only loosely homologous to asparagine synthetase (∼20% identity and ∼38% similarity),^30,31^ and no lasso peptide synthetase has ever been structurally characterized.

**Figure 2:**
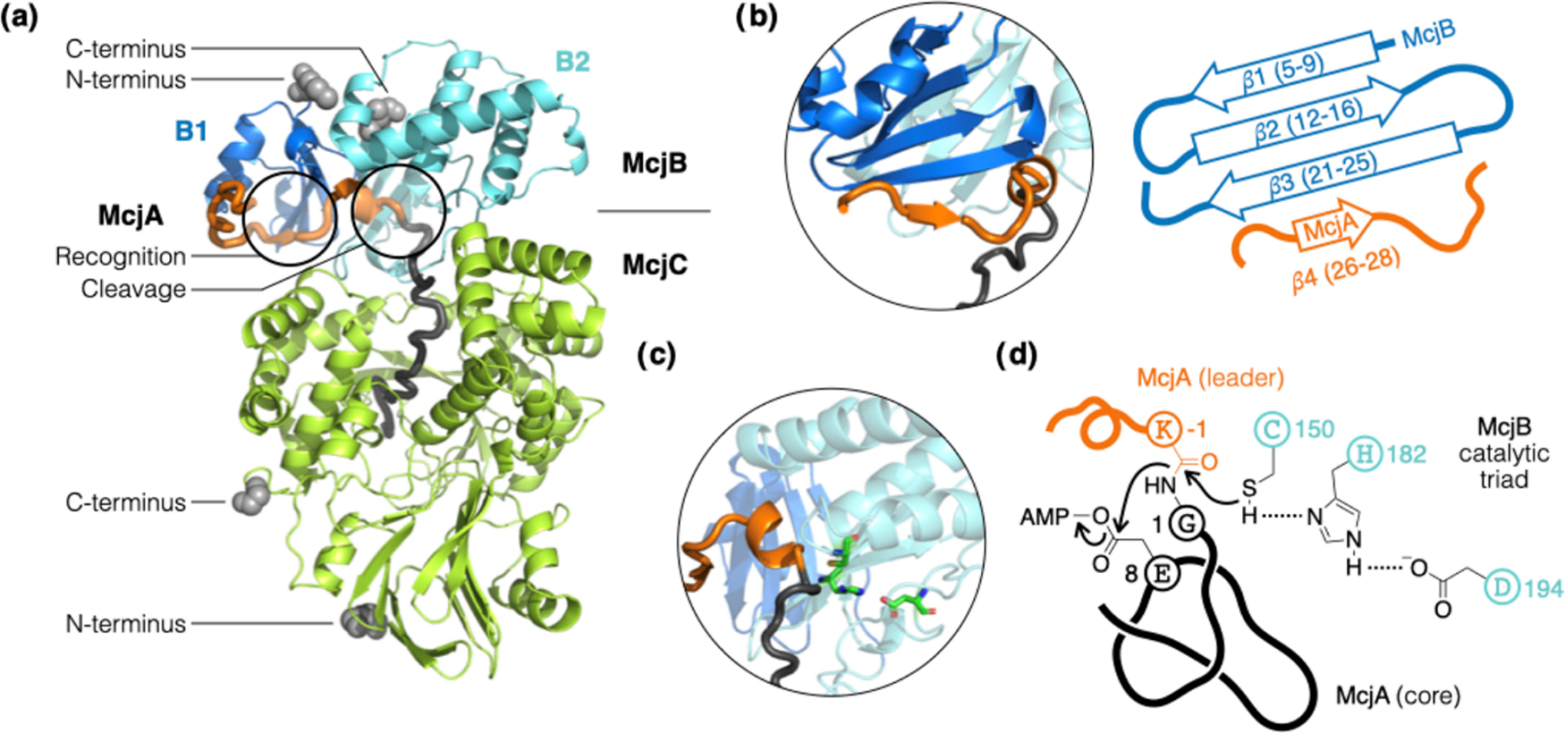
AF2 predicts the formation of a McjA/McjB/McjC ternary complex. (a) The leader peptide interacts mostly with McjB and the core peptide extends into a deep cavity in McjC. The first 13 residues of McjB are omitted for clarity. The N- and C-termini of McjB and McjC are shown as gray spheres; the rest of the structures are shown in cartoon and color-coded the same way as in Figure 1. (b) Key to leader peptide recognition is an antiparallel β-sheet in the B1 domain of McjB. A short segment of the leader peptide (McjA_(−12)to(−10)_) aligns along β3 as a fourth strand to extend the β-sheet. (c) The B2 domain of McjB is a protease whose Cys-His-Asp catalytic triad (shown in sticks) is poised to cleave the amide bond that connects the leader and the core peptides. (d) The speculative mechanism of MccJ25 maturation is shown, wherein McjB catalyzes leader cleavage, and McjC catalyzes isopeptide bond formation to entrap the tail of the core peptide. All protein structures were rendered by PyMOL.

McjA, McjB, and McjC were submitted as separate polypeptide sequences to AF2. Gratifyingly, the three proteins were predicted to form a ternary complex that shows structural features consistent with what is known about MccJ25 biosynthesis and RiPP maturation in general (Figure 2a). Specifically, the McjB predicted structure shows discrete N-terminal (B1) and C-terminal (B2) domains. The former displays a typical winged helix-turn-helix fold that harbors the RRE (Figure 2b); the latter shows a Cys-His-Asp catalytic triad typical of cysteine proteases, wherein the side-chain carboxylate, imidazole, and thiol moieties are aligned (Figure 2c). The thiol points directly at the scissile amide bond (McjA_K–1/G1_) that connects the leader and the core segments of the precursor (Figure 2d). The predicted structure also offered some new insights. For example, the active sites of McjB and McjC face each other and sandwich the leader peptide in between. The contact surface area between McjB and McjC is approximately 1000 Å^2^ and appears to be driven by a network of at least 10 hydrogen bonds (Figure S2).^32^ It is hypothesized that McjC stabilizes the core peptide in a folded conformation that restricts the motion of its own C-terminal tail when macrolactam formation takes place.^29,33^ While AF2 did predict the core peptide to extend into a deep cavity in McjC, the hypothesized prefolding mechanism of the core peptide was not evident in the predicted structure. The side-chain carboxylate of Glu8 (McjA_E8_) seems poised to be adenylated to form an active ester as it sits next to the ATP binding site in McjC (Figure S3).

Unless guided by structural information, modification of any of the three proteins will very likely disrupt the intricate coordination of this McjA/McjB/McjC biosynthetic complex and abolish MccJ25 production. On the other hand, protein engineering guided by (predicted) structural information shall have a higher rate of success. We designed a series of McjA, McjB, and McjC variants meant for testing critical structural features predicted by AF2 at various scales, from the orientation of interaction between proteins, to domain boundary within a protein, to specific contacts between individual amino acid residues. If a variant disrupted the McjA/McjB/McjC ternary complex, little or no MccJ25 would be produced. Conversely, robust MccJ25 production would suggest that AF2 presented a reasonable model for the structural feature probed by that particular protein variant. Protein variants were constructed by directly modifying the plasmid commonly used for MccJ25 production (pTUC202).^34^ Residues of interest were replaced and tested one at a time while leaving all other proteins unchanged (Table S2); MccJ25 production was determined by inspecting culture extracts in accordance with published procedures (Figure S4).^35,36^

### McjB/McjC Interface and Their Termini

The MBP (396 aa) is a commonly used tag to solubilize and stabilize recombinant proteins; it is almost twice as large as McjB (208 aa) and about three-fourths the size of McjC (513 aa). McjB and McjC are known to still be functional when the MBP is fused to their N-termini.^15^ For all of our protein variants (engineered McjA, McjB, or McjC in the pTUC202 vector expressed in *Escherichia coli*), culture extracts were first analyzed by MALDI-TOF MS, and those that showed the expected MccJ25 *m*/*z* signals were then subjected to an inhibition zone assay and LC quantitation. For the former, a 2-fold culture extract dilution series was prepared and spotted onto a bacterial lawn to compare the sizes of the growth inhibitory zones they generate. The latter entails integrating the MccJ25 peak area in an LC trace (Table 1), wherein caffeine was coinjected as the internal standard for quantitation.

**Table 1:** MccJ25 Production Yields for Various Protein Variants

construct	peak area^a^^,^^b^	yield^c^
WT	14 ± 2.3 × 10^3^	100%
MBP fusions		
MBP-McjB		ref (15)^d^
MBP-McjC		ref (15)^d^
McjB-MBP	8.5 ± 1.2 × 10^3^	61%
McjC-MBP	12 ± 0.6 × 10^3^	86%
McjB/McjC interface		
McjB_F67R_	82 ± 73	0.6%
circular permutations		
McjB_CP60/61_	51 ± 13	0.4%
McjB_CP70/71_	n.d.	
McjB_CP80/81_	610 ± 137	4.4%
split McjB		
McjB_S60/61_	n.d.	
McjB_S70/71_	n.d.	
McjB_S80/81_	5.8 ± 0.3 × 10^3^	41%
McjA/McjC recognition		
McjC_K337E_	1.1 ± 0.2 × 10^3^	8.1%
McjC_S440Y_	324 ± 86	2.4%
synthetic rescue		
McjA_T–2F_	n.d.	
McjA_T–2F_/McjB_F23T_	n.d.	
McjA_T–2M_	145 ± 2	1.0%
McjA_T–2M_/McjB_M108T_	755 ± 56	5.4%

a Area under the MccJ25 peak in an HPLC trace was integrated. All samples contained the same concentration of caffeine (100 μg/mL) as a quantitation standard.

b “n.d.” denotes not detected.

c Average yield relative to that of the WT; all assays were done in triplicate (*n* = 3).

d Rebuffat and co-workers reported the production of MccJ25 using these constructs in an in vitro reconstitution experiment.^15^

**Figure 3:**
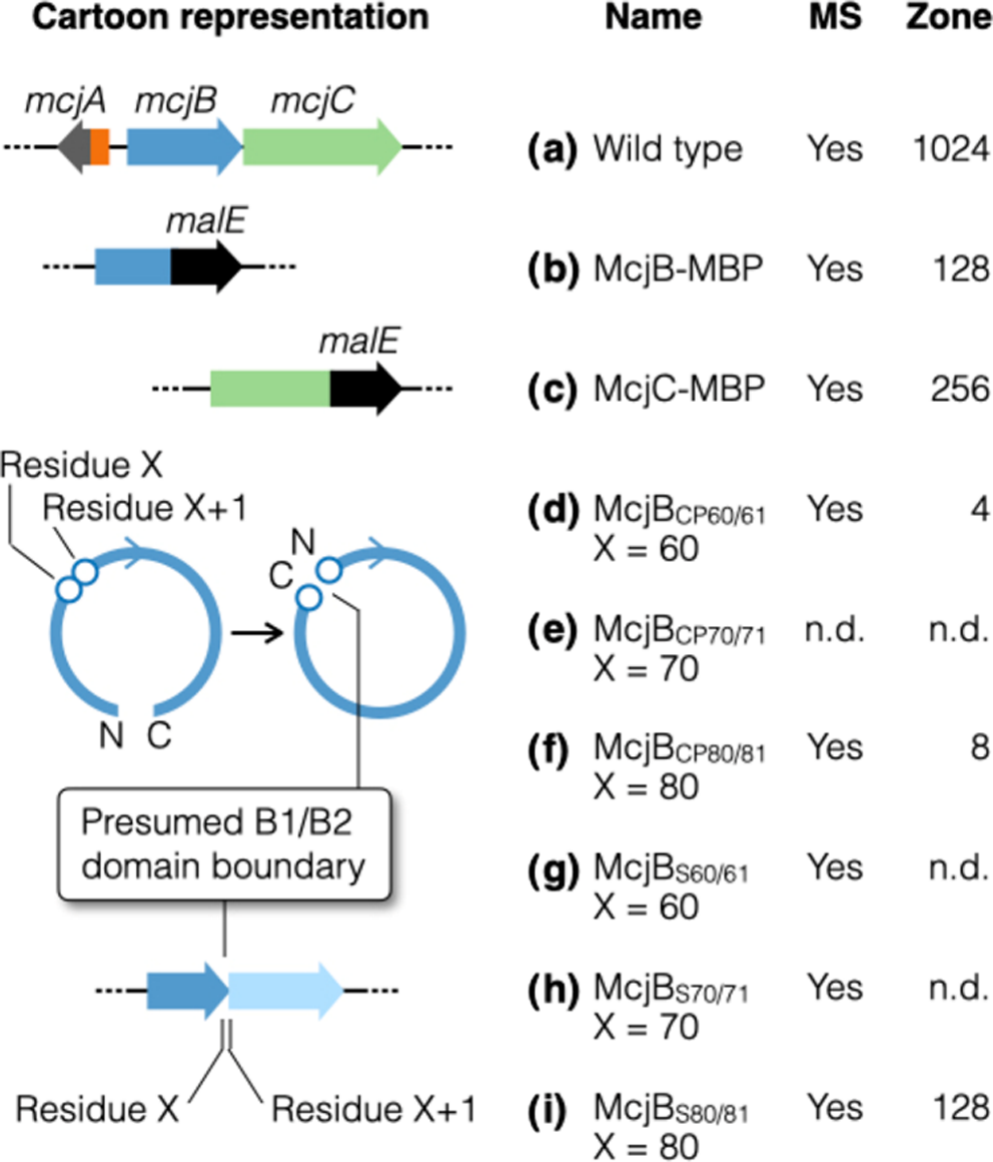
Series of protein variants designed based on AF2 predicted structures. Structural features of interest were tested one at a time, while all other proteins were kept unchanged. The inhibition zone assay is a semiquantitative assessment of MccJ25 production. The WT was assigned an arbitrary score of 1024; the variants were scored by recording and comparing to the WT their most diluted extract that generated a growth inhibition zone with a diameter of 1.0 cm (see the Supporting Information for details). All assays were done in triplicate (*n* = 3); “n.d.” indicates that it was not detected. Extracts that resulted in detectable inhibition zones were further quantitated by HPLC peak integration (Table 1). The following variants were evaluated: (a) Wild type; (b, c) McjB and McjC with a C-terminal MBP fusion. Note that N-terminal MBP fusions of these proteins have already been reported (ref (15)); (d–f) McjB_CPX/X+1_ denotes a CP variant with its original residues X and X+1 now serving as the new C- and N-termini, respectively; (g–i) McjB_SX/X+1_ denotes splitting McjB into two proteins B1 and B2, which comprise the original residues 1-to-X and X+1-to-208, respectively.

We constructed C-terminal MBP fusions of McjB and McjC and tested them, one at a time, in a background of otherwise native BGC. The MccJ25 production of these constructs were compared to the WT MccJ25 BGC via both qualitative and quantitative methods described above (Figure 3a–3c). These constructs showed only a slight decrease in MccJ25 production yield compared with the native enzymes (Table 1), suggesting that having a sizable protein tag on the C-terminus of either McjB and McjC does not interfere with the formation of a catalytically proficient enzyme complex. The AF2 predicted structure of the McjA/McjB/McjC ternary complex is in line with these observations, which shows the N- and C-termini of both McjB and McjC all pointing away from their interaction interface.

We also wanted to probe the McjB/McjC interface. As a residue on the protein surface and far away from the catalytic triad, variants of McjB_F67_ are expected to still be proficient proteases. However, AF2 predicted that this residue (McjB_F67_) engages in a cation-π interaction with McjC_R438_ and also lies at the heart of a network of hydrogen bonds at the McjB/McjC interface (Figure S2). The McjB_F67R_ variant, wherein the Phe aromatic ring is replaced by the positively charged guanidinium in Arg, should abolish both types of noncovalent attraction described above. The resulting McjB/McjC interaction would be much weaker and result in decreased MccJ25 production. MccJ25 production for the McjB_F67R_ construct did turn out to be much lower compared to that for the WT (0.6%, Table 1 and Figure S5a).

### Circular Permutation of McjB

**Figure 4:**
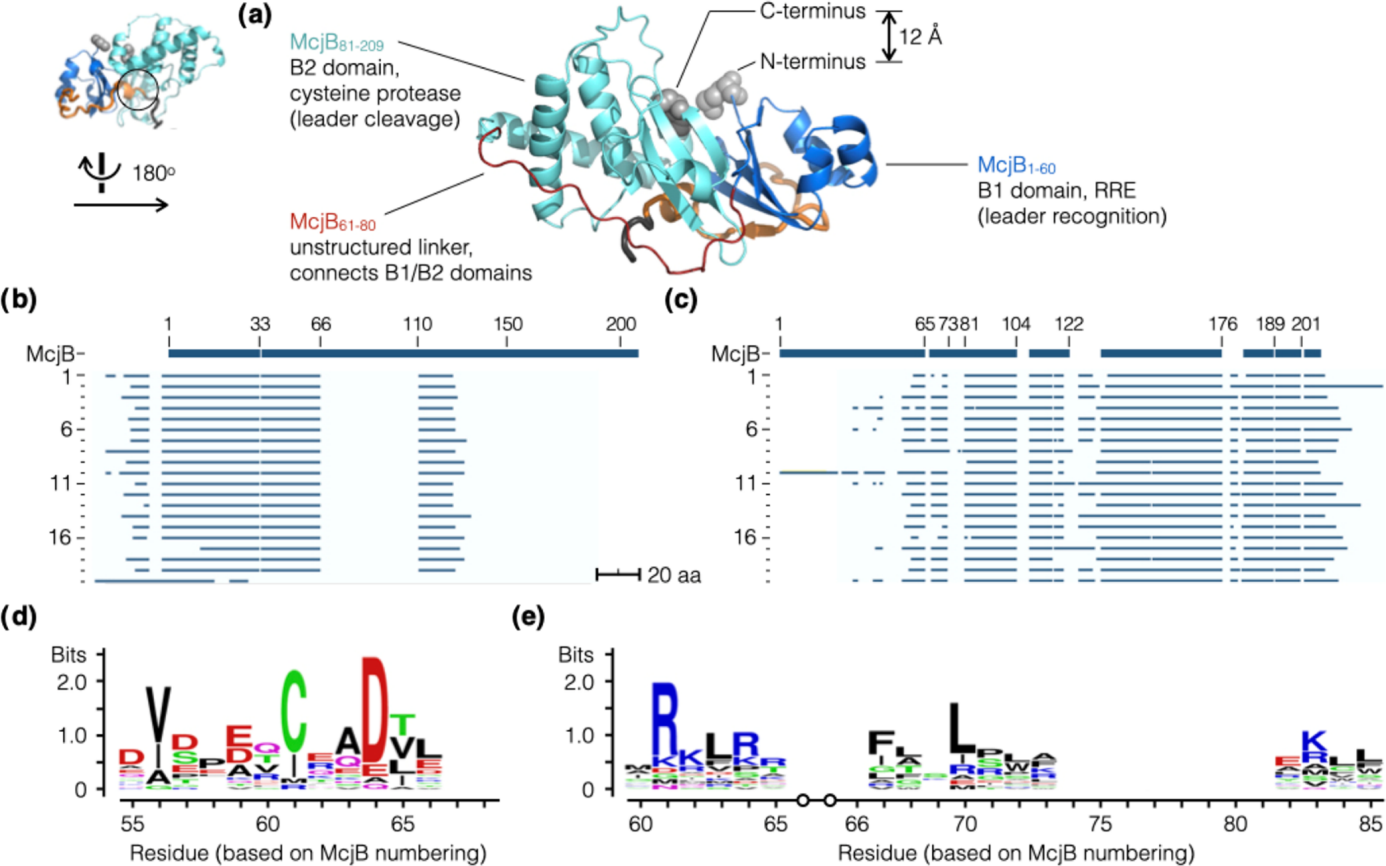
Identifying the B1/B2 domain boundary in McjB. (a) AF2 predicted McjB to fold into discrete domains, B1 (RRE, residues 1 to 60, blue) and B2 (protease, residues 81 to 208, cyan), that are connected by a linker with no apparent secondary structure (residues 61 to 80, red). Its N- and C-termini are located in close proximity and prompted us to test a series of circularly permutated and split variants of McjB. (b, c) We compiled a collection of phylogenetically diverse lasso peptide BGCs (20) that contain separate B1 and B2 proteins. The sequences of these B1 (b) and B2 proteins (**c**) were aligned to McjB; logo plots were generated for sequence alignments of B1 (d) and B2 (e). These results corroborated the predicted AF2 structure, suggesting that the domain boundary is likely somewhere in the 61–80 region.

Upon closer examination of the AF2 predicted structure, we noticed that the N- and C-termini of McjB are spatially very close to each other (Figure 4a). Their Cα atoms are merely 12.3 Å apart. This inspired us to explore the possibility of circular permutation (CP), which can be viewed as if a string was circularized and then cut open at a different site.^37,38^ In a CP protein, the N- and C-termini of the native protein are connected (usually via a short linker) and new termini are created elsewhere in the native sequence.^39^ The resulting CP protein variant has two stretches of amino acid sequences that are each identical to part of the native protein, yet their connectivity is rearranged. To generate McjB CP variants, we used a short flexible spacer (GGSSGG) to link its original termini and chose the positions of the new termini based on sequence analysis and the AF2 predicted structure.

While McjB is a single polypeptide, the B enzymes of Gram-positive bacterial lasso peptide BGCs often exist as separate B1 and B2 proteins.^26^ The B1/B2 domain boundary in McjB would be the ideal new terminus if a CP variant was to be generated. A collection of 20 phylogenetically diverse lasso peptide BGCs that harbor discrete B1 and B2 ORFs was compiled (Table S3).^40^ The B1 proteins and the B2 proteins were then aligned separately to McjB. All B1 and B2 proteins were aligned to the N- and C-termini of McjB, respectively. In the 61-to-80 segment (numbering based on the McjB sequence), sequence alignments showed multiple sharp edges (Figure 4b,4c) and logo plots identified multiple highly conserved residues (Figure 4d,4e). These results suggest that residues 61 to 80 in McjB are likely where its B1/B2 domain boundary is located. The AF2 predicted structure corroborated this notion, showing two discretely folded domains connected by a stretch of amino acid residues (59 to 83) without a well-defined secondary structure.

Based on the above analysis, we generated three variants, McjB_CP60/61_, McjB_CP70/71_, and McjB_CP80/81_, wherein McjB_CPX/X+1_ denotes a CP variant with new C- and N-termini at residue X and X+1, respectively (Figure 3d–3f). Out of the three constructs, two showed MccJ25 production, wherein the yield of the McjB_CP80/81_ construct was about an order of magnitude higher than that of McjB_CP60/61_ (Table 1). These results are the first reports of a circularly permutated lasso peptide biosynthetic enzyme.

### Splitting McjB into Two B1 and B2 Proteins

Marahiel and co-workers showed that the B enzyme of the rubrivinodin BGC (RugeB) can be split into B1 and B2 proteins and still produce the same lasso peptide, albeit at a much lower yield.^19^ In the case of MccJ25 production, if McjB were to be split into two proteins, the split site must be chosen carefully to ensure that the intricate interaction is preserved, and the resulting B1 and B2 proteins can still form a proficient enzyme complex with McjC to catalyze McjA maturation. We again consulted sequence alignment results and the AF2 predicted structure. To split McjB, we inserted in *mcjB* a 32-nucleotide spacer that includes a stop codon (TAA), a short spacer, a ribosome binding site (AAGGAG), and a start codon (ATG). Three constructs were generated (McjB_S60/61_, McjB_S70/71_, and McjB_S80/81_), wherein McjB_SX/X+1_ denotes splitting the native McjB to create a B1 protein that ends at residue X and a B2 protein that starts at residue X + 1 (Figure 3g–3i). Out of the three constructs, one variant showed robust production (McjB_S80/81_), yielding slightly less than half as much of MccJ25 (41%) compared to the WT (Table 1). These data again suggest that the AF2 predicted structure of the McjA/McjB/McjC complex is accurate enough to help guide the design of protein variants.

### Disrupting McjA Recognition by McjC

**Figure 5:**
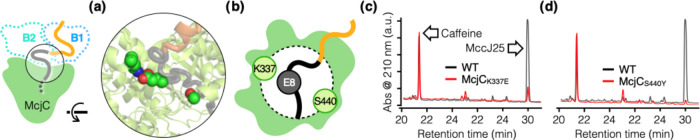
McjC residues proximal to McjA_E8_ are important for McjA recognition. (a) Top view of the rim of the McjC cavity (McjB was omitted for clarity), which accommodates the core of McjA and is hypothesized to prefold it into a conformation poised to form the threaded structure upon Gly1-Glu8 isopeptide bond formation. (b) Cartoon illustration of McjA and the McjC cavity. McjC_K337_ and McjA_E8_ form a salt bridge (2.9 Å apart), and McjC_S440_ is on the opposite side of the rim. (c, d) McjC_K337E_ and McjC_S440Y_ variants were meant to disrupt their noncovalent interaction with McjA_E8_, which was expected to weaken McjA/McjC interaction and decrease MccJ25 yield. HPLC analysis (representative trace) showed that McjC_K337E_ and McjC_S440Y_ produced much lower amounts of MccJ25 compared to the WT (8.1 and 2.4%, respectively). Experiments were done in triplicate (*n* = 3, Table 1); caffeine was coinjected as a quantitation standard.

The predicted McjC structure showed a central cavity that accommodates the core peptide (McjA_1–21_) and is consistent with the hypothetical prefolding mechanism for MccJ25 biosynthesis. Presumably, McjC stabilizes the core peptide in a conformation, wherein McjA_G1-E8_ wraps around the tail such that the threaded structure characteristic of MccJ25 is generated upon formation of the Gly1-Glu8 isopeptide bond to create the macrolactam ring (Figure 1). AF2 provided insights into how McjC controls the position and orientation of the McjA_E8_ side-chain carboxylate (Figure 5a,5b), both of which are of paramount importance in such a biosynthetic mechanism. There is an apparent salt bridge between the McjC_K337_ side-chain amine and the McjA_E8_ side-chain carboxylate; the two functional groups are only 2.9 Å apart in the predicted ternary complex structure. We constructed the McjC_K337E_ variant to probe this predicted feature, wherein the Lys-to-Glu substitution was expected to eliminate the salt bridge interaction and disrupt McjA recognition by McjC. The McjC_K337E_ variant produced MccJ25 at a much lower yield compared to the WT (8.1%, Table 1 and Figure 5c). In addition, the McjC_S440Y_ variant was generated to probe the effect of steric hindrance. This residue is on the rim of the McjC cavity and is spatially close to McjA_E8_. Replacing Ser with Tyr, a much larger residue that can still engage in hydrogen bonding, is expected to negatively impact McjA/McjC interaction. Indeed, this construct produced MccJ25 at a much lower yield compared to that of the WT as well (2.4%, Table 1 and Figure 5d).

### Synthetic Rescue of McjA Recognition by McjB

Perhaps the ultimate test of the AF2 predicted structure of the McjA/McjB/McjC ternary complex is to use it to pinpoint the molecular basis of the McjA/McjB interaction. Link and co-workers showed that truncating up to 28 N-terminal residues of the leader peptide still led to detectable MccJ25 production,^41^ suggesting that leader peptide recognition by McjB for the most part can be attributed to the rest of the eight C-terminal residues. The importance of Thr(−2), which refers to the second to the last residue of the leader peptide (McjA_T–2_), was also well established.^42^ Mutations at this position often negatively impact MccJ25 production. For example, the T-2 M variant yielded only ∼1% of the amount of MccJ25 compared with the WT, and the T-2F variant failed to produce any detectable amount of MccJ25. In addition, a large-scale bioinformatic analysis of lasso peptide BGCs revealed that residues at this penultimate “minus-two” position of the leader peptide is highly conserved, of which 94% are Thr.^26^ However, the binding pocket in McjB that is involved in McjA (leader peptide) recognition has not been identified.

**Figure 6:**
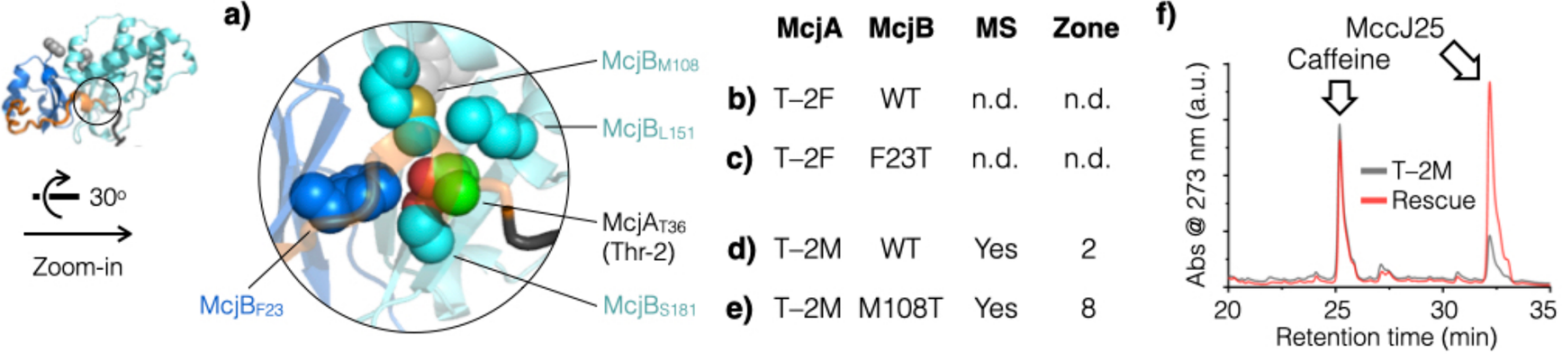
Examine leader peptide recognition in detail. (a) AF2 predicted the penultimate Thr(−2) of the leader peptide (McjA_T–2_) to fit snugly in a binding pocket made of four McjB residues (shown in spheres). Two synthetic rescue pairs were designed. (b, c) The McjA_T–2F_ variant did not produce any detectable amount of MccJ25 and the McjB_F23T_ mutant failed to rescue it. (d, e) The McjA_T–2M_ variant produced an extremely low amount of MccJ25 and the compensatory McjB_M108T_ mutation successfully increased the MccJ25 yield by more than 5-fold based on HPLC quantitation (Table 1). (f) Representative HPLC traces showing that the TM construct (the synthetic rescue pair McjA_T–2M_/McjB_M108T_) greatly increased MccJ25 production compared to the McjA_T–2M_ variant. HPLC analysis was done in triplicate (*n* = 3); caffeine was coinjected as a quantitation standard.

In the AF2 predicted structure, the side chain of Thr(−2) fits snugly in a binding pocket formed collectively by four McjB residues, including one from the B1 domain (F23) and three from the B2 domain (M108, L151, and S181) (Figure 6a). The increase in side-chain size in both of the aforementioned McjA variants (T-2F and T-2M) must have turned what used to be a snug fit into steric clashes, compromised McjA precursor recognition by McjB, and resulted in diminished MccJ25 production (or a total loss thereof). It may be possible to restore MccJ25 production for these McjA variants by introducing compensatory mutations into McjB to expand the binding pocket, a procedure termed “synthetic rescue”. We hypothesized that when an X-to-Y exchange in the substrate makes it too big to fit in the binding pocket, a concomitant Y-to-X substitution in the binding pocket should alleviate the steric clash to restore substrate/enzyme interaction. Based on this simple design rationale, we generated two pairs of constructs – McjA_T–2F_/McjB_F23T_ (TF) and McjA_T–2M_/McjB_M108T_ (TM) (Figure 6b–6e). Even though the TF construct failed to boost MccJ25 production, the TM construct increased MccJ25 production yield by more than 5-fold (Figure 6f and Table 1). These results suggest that the AF2 predicted structure of the ternary complex is a reasonable model to identify individual residues involved in leader peptide recognition.

## Discussion

MccJ25 is one of the first lasso peptides discovered.^7^ While many more examples have been found and studied since, MccJ25 is unique even among this family of natural products. Specifically, the two enzymes that catalyze MccJ25 maturation (McjB and McjC) depend on the presence of each other to function.^15^ This observation, together with the AF2 predicted structure reported herein, hints at an intriguing concerted biosynthetic process; however, this notion has thus far remained a speculation that awaits further experimental support (Figure 2d). Despite immense interest in MccJ25 over the past three decades,^8^ structural information on the MccJ25 biosynthetic machinery is still unavailable and molecular details of how the “threaded lasso” configuration is constructed remain elusive. AF2 was used to predict the structure of the McjA/McjB/McjC ternary complex, and our experimental results support all of the predicted structural features we tested, including protein orientation, domain boundaries, and contact between select amino acids.

Our CP McjB variants represent the first attempt at engineering a lasso peptide biosynthetic enzyme in this manner. Interestingly, we identified four lasso peptide B enzymes out of a database of 1,619 (0.2%) that appear to have an N-terminal protease domain (B2) and a C-terminal RRE (B1) (Table S4).^26^ Sequence alignment and structure prediction by AF2 both supported this domain assignment; i.e., these B enzymes naturally have an “inverse” domain arrangement (Figures S6 and S7). If B1 and B2 started out as separate proteins, based on our CP results and the few examples that exist in nature, there are viable ways to join them, in either order, to create a fused B protein that is functional. However, we are unaware of any evolutionary or genetic mechanisms that would bias the direction of a hypothetical B1 and B2 protein fusion event to such an extreme ratio (99.8% (=1 – 0.2%)). Seeing only very rare cases of “inverse” domain arrangement in nature (0.2%) would seem to suggest that the B1 and B2 proteins of today resulted from an ancient B protein splitting into two during the course of evolution. This notion is an interesting speculation that is worth testing in the future.

The interaction between McjA and McjB warrants further discussion. Large-scale sequence analysis and protein structural studies point to a general mode of leader recognition in RiPP biosynthesis.^26^ The leader peptide first binds to the RRE, which has a winged helix-turn-helix fold, with high affinity and specificity.^17^ Central to this interaction is a β-sheet in the RRE with three antiparallel strands (β1–3), and all RREs (15) structurally characterized to date show this feature (Table S1). Based on the AF2 predicted structure, the three strands correspond to residues 5–9 (β1), 12–16 (β2), and 21–25 (β3) of McjB, and the leader peptide aligns itself along the edge of β3 of McjB to form a fourth strand (β4, McjA_(−12)to(−10)_) to extend the β-sheet (Figure 2b). Approximately 6 to 7 residues in the therbactin and the fusilassin precursors interact with their respective RRE (TbiB1 and TfuB1),^18,27^ whereas only three McjA residues (−12, −11, and −10) appear to be involved. The latter interaction is likely much weaker in comparison, which, coupled with McjA_(−37)to(−13)_ showing no obvious interaction with McjB in the predicted structure, explains why much of the McjA N-terminal sequence is dispensable (McjA_(−37)to(−9)_).^41^

This observation also implies that the few residues immediately upstream of the core peptide (McjA_(−8)to(−1)_) must play an outsized role in interacting with McjB. We therefore directed our attention to the highly conserved Thr(−2) residue (McjA_T–2_). Previous studies showed that mutations at this residue negatively impact MccJ25 production; e.g., the McjA_T–2M_ variant yielded less than 1% of MccJ25 compared to the WT. This mutation presumably made the residue at the key “minus-two” position too bulky to fit in the McjB binding pocket. Using the AF2 predicted structure as a guide, our synthetic rescue construct (the introduction of a compensatory McjB_M108T_ mutation) partially restored the McjA/McjB interaction, evidenced by an increase in yield by more than 5-fold. Interestingly, AF2 still predicted the formation of a McjA/McjB/McjC ternary complex for both the T-2F and T-2 M constructs, wherein the Phe and Met residues that replaced Thr showed no obvious clashes with the McjB binding pocket. This result suggests that while AF2 can often predict individual protein structures fairly accurately, it sometimes fails to reflect the consequences of subtle changes, especially when it comes to protein–protein interactions.

The unstructured linker segment (McjB_61–80_) is part of the McjB/McjC interface (Figure 1a). To construct our CP and split McjB variants, an extra pair of charges is generated in this region: a net positive and a net negative charge associated with the new N- and C-termini, respectively. Since the McjB_61–70_ segment is in direct contact with McjC and residues 80 and 81 are off to the side, it is not surprising that the McjB_CP80/81_ and McjB_S80/81_ variants showed the highest MccJ25 yields among our CP and split McjB constructs, respectively (Table 1). These observations suggest that a proficient complex that catalyzes MccJ25 maturation can still form when McjB is split at residue 80/81. In fact, when McjA/McjB_1–80_/McjB_81–208_/McjC were submitted as four separate polypeptides, AF2 predicted a quaternary complex nearly identical to the native McjA/McjB/McjC ternary complex (Figure S8). Future engineering of B enzymes should therefore take this into consideration and avoid amino acid changes that are part of the McjB/McjC interface.

## Conclusions

Altogether, the data presented herein suggest that the structure of the McjA/McjB/McjC ternary complex predicted by AF2 is a reasonable model and can serve as a starting point toward understanding the structural basis of MccJ25 biosynthesis. In addition to presenting a structural model of leader peptide interaction with McjB, AF2 also predicted the core peptide to extend into a deep cavity in McjC. As no lasso peptide synthetase has ever been structurally characterized, it is not surprising that AF2 fell short of informing us the exact molecular details of the presumed McjC-stabilized prefolded conformation of the core peptide. Enzymes that catalyze the formation of such a supramolecular structure are currently beyond the reach of computation and artificial intelligence. This final knowledge gap in lasso peptide biosynthesis will have to be filled by experiments and human intelligence.

## Supporting Information Available

Materials and methods; workflow for MccJ25 production, QC, and MS confirmation; supporting figures and tables (PDF)
AF2 predicted structure (PDB)

## Supplementary Material

cb4c00251_si_001.pdf

cb4c00251_si_002.pdb
