# Supplementary material for: Structure Prediction and Protein Engineering Yield New Insights into Microcin J25 Precursor Recognition
Source: ACS Chem Biol. 2024 Aug 20;19(9):1982–90. doi: 10.1021/acschembio.4c00251 (PMC11420955; doi:10.1021/acschembio.4c00251)
Supplement: Supplementary file 1 — cb4c00251_si_001.pdf [file cb4c00251_si_001.pdf]

Structure prediction and protein engineering yield new insights into microcin J25 precursor recognition

Hui-Ni Tan[1], Wei-Qi Liu[1], Josh Ho[1], Yi-Ju Chen[1], Fang-Jie Shieh[1], Hsiao-Tzu Liao[2], Shu-Ping Wang[3], Julian D. Hegemann[4], Chin-Yuan Chang[2], and John Chu[1]*

[1]Department of Chemistry, National Taiwan University, Taipei City 106319, Taiwan. [2]Department of Biological Science and Technology, National Yang Ming Chiao Tung University, Hsinchu 300193, Taiwan. [3]Institute of Biomedical Sciences, Academia Sinica, Taipei City 115201, Taiwan. [4]Helmholtz Institute for Pharmaceutical Research Saarland, Helmholtz Centre for Infection Research, Saarland University Campus, 66123 Saarbrücken, Germany.

Correspondence (*): John Chu (johnchu@ntu.edu.tw)

Table of Contents **Page**

Materials and Methods

- Reagents, media, and strains 2
- MBP-McjC production and analysis 2
- AlphaFold2 structure prediction 3
- MccJ25 production 3
- HPLC analysis (quality check) 3
- Growth inhibition zone assay 4
- HPLC analysis (quantification) 4
- B enzymes with inverse domain arrangement 4

Supplementary Figures

- ***Figure S1*** | MBP-McjC forms high molecular weight aggregates 6
- ***Figure S2*** | Hydrogen bonds at the McjB/McjC interaction interface 7
- ***Figure S3*** | ATP binding site in McjC 8
- ***Figure S4*** | The workflow for MccJ25 production, QC, and MS confirmation 9
- ***Figure S5*** | $McjB_{F67}$ plays an important role in McjB/McjC interaction 10
- ***Figure S6*** | B enzymes with inverse domain arrangement (sequence alignment) 11
- ***Figure S7*** | B enzymes with inverse domain arrangement (AF2 structure predictions) 12
- ***Figure S8*** | The $McjB_{S80/81}$ construct is predicted to form a quaternary complex 13

Supplementary Tables

- ***Table S1*** | Structurally characterized RiPP biosynthetic "B" enzymes 14
- ***Table S2*** | DNA primers used in this study 15
- ***Table S3*** | Representative B proteins for McjB sequence alignment (Figure 4) 17
- ***Table S4*** | List of B enzymes with inverse domain arrangement 20

References 21

MATERIALs and METHODs

Reagents, media, and strains

All engineered protein variants described herein were derived from pTUC202.[1] The pTUC202 plasmid contains the wild-type (WT) microcin J25 (MccJ25) biosynthetic gene cluster (BGC), which encodes four genes *mcjA*, *mcjB*, *mcjC*, and *mcjD*. DNA primers for cloning and sequencing were purchased from Genomics BioSci & Technology Company (Taiwan) and are listed in Table S1. Reagents and kits for site-directed mutagenesis (E0554) and Gibson assembly (E2621) were purchased from New England Biolabs (USA). Other reagents were purchased from BioShop Canada Inc. (Canada). Constructs described herein have all been verified by Sanger sequencing by Genomics BioSci & Technology Company (Taiwan). Cloning was carried out in *Escherichia coli* DH5α grown in Luria-Bertani (LB) medium supplemented with chloramphenicol (25 μg/mL); MccJ25 production was performed in *E. coli* BL21(DE3) grown in M9 medium supplemented with chloramphenicol (25 μg/mL); growth inhibition zone assays were carried out against *E. coli* MG1655.

MBP-McjC production and analysis

The *mcjC* gene was cloned into the pET-28a vector and expressed in *E. coli* BL21(DE3) as an N-terminal His-tagged recombinant protein. An overnight starter culture (3 mL) was used to inoculate fresh LB medium (1 L) supplemented with 50 μg/mL of kanamycin. When the cell density reached OD_{600} 0.5, McjC expression was induced by adding isopropyl-β-D-1-thiogalactopyranoside to a final concentration of 1 mM. Cells were grown at 16 ^{o}C for 16 h after induction and harvested by centrifugation at 6,300 × g at 4 °C for 30 min. The cell pellet was resuspended in lysis buffer (500 mM NaCl and 20 mM Tris, pH 8.0), disrupted by sonication, and then centrifugated again at 16,700 × g at 4 °C for 30 min to spin down the cell debris. The McjC recombinant protein was purified using Ni-NTA affinity chromatography [HisTrap FF (Cytiva, USA)] and size-exclusion chromatography [HiLoad Superdex 16/600 200 pg (Cytiva, USA)] on a NGC Chromatography Systems (Bio-Rad, USA).

Molecular weight (MW) of the His_6-McjC aggregate was estimated by size-exclusion chromatography [Superdex 200 10/300 GL (Cytiva, USA)]. Pure His_6-McjC was dissolved in 0.5 mL of loading buffer (100 mM NaCl and 20 mM Tris, pH 8.0) and injected. The flow rate was fixed at 0.45 mL/min and the result was compared to a set of commercially available Gel Filtration

Standards (Bio-Rad, USA). The estimated molecular weight of the His_6-McjC oligomer is 803.6 kDa (monomer = 58.7 kDa).

AlphaFold2 structure prediction

We used the default settings on Google Colaboratory (ColabFold v1.5.5) for AlphaFold2 (AF2) protein structure prediction.[2] The predicted protein structures were presented after rending by using the PyMOL Molecular Graphics System, v.2.0 (Schrödinger LLC, USA). The structures of McjA, McjB, and McjC were first predicted individually. Then, by submitting two protein sequences at the same time separated by a colon (:), the predicted structures all pairwise combinations of these proteins (McjA/McjB, McjB/McjC, and McjA/McjC) were obtained. Ten hydrogen bonds were identified at the McjB/McjC interaction interface (Figure S2),[3] and the side-chain carboxylate of Glu8 in McjA was predicted to be positioned next to the ATP binding site in McjC (Figure S3). Finally, three proteins were submitted together. The predicted structure of the McjA/McjB/McjC ternary complex was consistent with those obtained individually or as pairwise combinations.

MccJ25 Production

Procedure for MccJ25 production is based on a previous publication[4] and slightly modified as follows. A single *E. coli* BL21(DE3) colony carrying pTUC202 was inoculated into LB medium (3 mL) supplemented with chloramphenicol (25 μg/mL) and grown at 37 °C overnight. This starter culture was diluted to OD_{600} 0.1 in M9 medium (100 mL) supplemented with chloramphenicol (25 µg/mL) and grown at 37 °C for 4 days. Cells were removed by centrifugation on the 5th day, and the supernatant was extracted with 1-butanol (200 mL). The organic layer was collected and dried under reduced pressure. The crude extract was either lyophilized for long-term storage (-20 ^{o}C) or redissolved in H_2O (12 mL) for quality check by HPLC and mass spectrometry (MS) analysis (Figure S4). MccJ25 productions by *E. coli* carrying the WT or the engineered plasmid (containing variants of McjA, McjB, or McjC) were carried out in the exact same way.

HPLC Analysis (quality check)

HPLC analysis was performed on a Waters Instrument (996 UV detector, 600 pump and controller) equipped with an analytical SHARPSIL-U C18 column (250 $\times$ 4.6 mm ID, 100 Å, 5 μm) using a two-solvent system. Water and acetonitrile supplemented with 0.1% (v/v) formic acid were used as the mobile phase, designated as solvent A and B, respectively. The following program was used: 0 – 5 min (10%B, isocratic), 5 – 25 min (10 to 50%B, linear gradient), 25 – 26 min (50

to 90%B, linear gradient), 26 – 30 min (90%B, isocratic). The flow rate was fixed at 1 mL/min and the absorbance at 210 nm was recorded. MccJ25 typically eluted at t_R = 26.3 ± 0.5 min; confirmation by MALDI-TOF MS (micrOTOF-QII, Bruker) was performed for every HPLC run. The sodium and potassium adduct $[M+Na]^+$ and $[M+K]^+$ were usually observed in MS (calculated m/z = 2129.55 and 2145.40, respectively).

Growth Inhibition Zone Assay

We used the size of growth inhibition zone as a semi-quantitative assessment of the level of MccJ25 production. LB medium was inoculated by a single colony of *E. coli* MG1655, grown overnight at 37 °C, and diluted 100-fold into 10 mL of molten LB agar. The bacteria / molten agar mixture was then poured into a petri dish (*d* = 10 cm), and left to solidify as it cools down in a sterile biosafety hood. Crude extracts obtained from 100 mL of MccJ25 production culture were resuspended in 1 mL of aqueous dimethyl sulfoxide (10%, v/v), which was used as the stock solution to generate a 2-fold dilution series in LB medium. MccJ25 at each dilution was spotted (5 μL) onto the LB agar embedded with *E. coli* MG1655 and incubated at 37 °C for 16 h. The most diluted extract to generate a growth inhibition zone with a diameter of 1 cm was recorded. All variants were compared to the WT, which was assigned an arbitrary score of 1,024.

HPLC analysis (quantification)

The instrument and solvent system are the same as described above (see *HPLC analysis, quality check*). A slightly different program was used: 0 – 5 min (1%B, isocratic), 5 – 30 min (1 to 50%B, linear gradient), 30 – 35 min (50 to 90%B, linear gradient), 35 – 39 min (90%B, isocratic). The flow rate was fixed at 1 mL/min and the absorbance at 273 nm was recorded; MccJ25 typically eluted at 32.5 ± 0.3 min. Caffeine was premixed with the crude extract (100 μg/mL, final concentration) and co-injected as a quantitation standard.

B enzymes with inverse domain arrangement

Two out of three circular permutation variants we constructed showed robust MccJ25 production, and one of them ($McjB_{CP80/81}$) can even generate milligrams of material per liter of culture extract. In light of these results, it is logical to ask whether natural lasso peptide biosynthetic B enzymes may present such an "inverse" domain arrangement, *i.e.*, having an N-terminal protease domain (B2) and a C-terminal RRE (B1). Mitchell and co-workers compiled a

database of 5,193 lasso peptide BGCs, the largest lasso peptide BGC to date, wherein 1,619 of them encode a fused B enzyme.[5] Ten pairs of discrete B1/B2 proteins of diverse phylogenetic origin were chosen as the reference sequences[6] and aligned to each of the 1,619 fused B enzymes. The vast majority of them (1,615) showed the typical B1-B2 arrangement, whereas four candidate B enzymes (Table S4), all associated with Actinomycete species, appeared to have an inverse domain arrangement. The N- and C-terminal sequences of these four candidate B enzymes aligned to the reference sequences for B2 and B1, respectively (Figure S5).

Next, we used AF2 to predict the structures of these candidate B enzymes, which turned out to corroborate the sequence alignment results (Figure S6). Their N-terminal domains all have a Cys-His-Asp catalytic triad typical of cysteine proteases and C-terminal domains all show the winged helix-turn-helix (wHTH) fold typical of RREs. RREs whose structures have been determined, TbiB1[7] and TfuB1 (FusB1),[8] are highly similar to and can be superimposed with the C-terminal domains of these four candidate B enzymes. These results strongly support the notion that we have identified four lasso peptide B enzymes that are opposite in domain arrangement to the vast majority proteins in this family.

SUPPLEMENTARY FIGUREs

Figure S1 | MBP-McjC forms high MW aggregates.

a) SDS-PAGE of pure His_6-McjC. Lane 1 and 2 are the marker and purified McjC, respectively. The molecular weight of the His_6-McjC monomer calculated based on its sequence is 58.7 kDa and is consistent with the mobility observed on a denaturing gel. **b)** The MW calibration curve was obtained by injecting the Gel Filtration Standards purchased from Bio-Rad (USA) (black datapoints). The apparent MW of the His_6-McjC aggregate (red datapoint) is estimated to be 803.6 kDa, corresponding to an aggregate of 10+ monomers (*n* = 13.7).

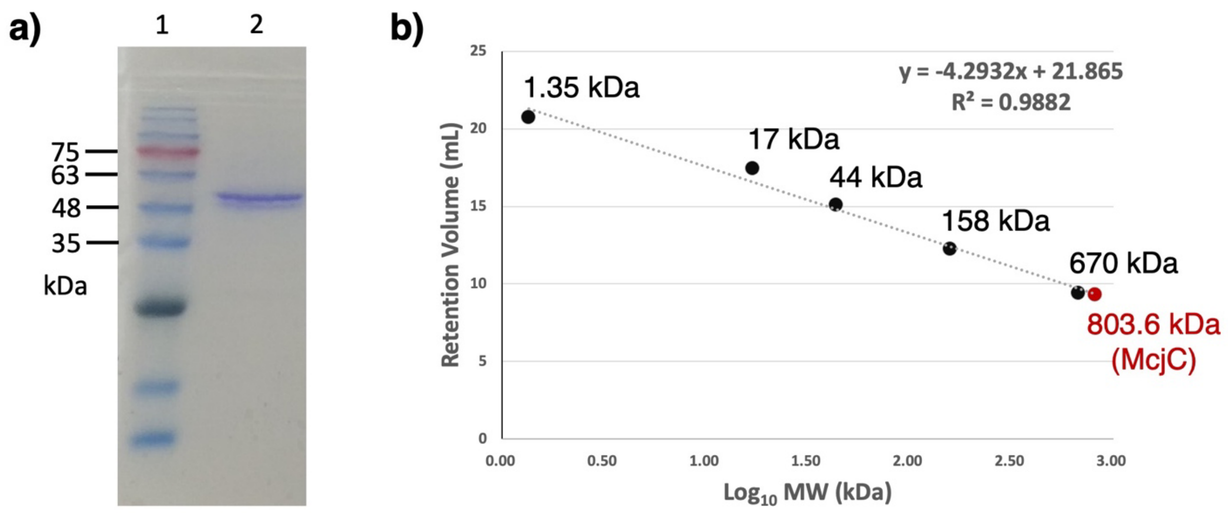

Figure S2 | Hydrogen bonds at the McjB/McjC interaction interface

Ten hydrogen bonds were identified at the McjB/McjC interface based on the AF2 predicted structure[2] and algorithm developed by Krissinel and Henrick.[3] Four residues (T65, S66, F67, and R71) within the unstructured linker region ($McjB_{61-80}$) that connects the B1 and B2 domains are involved in hydrogen bonding.

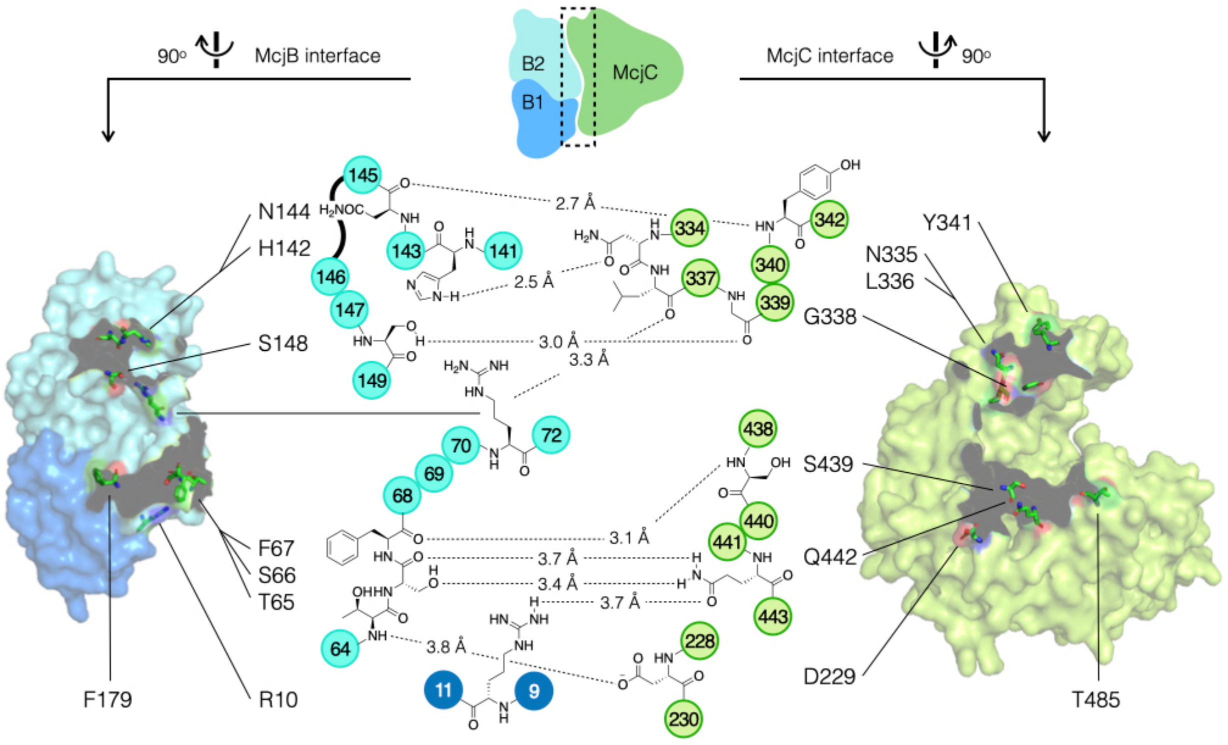

Figure S3 | ATP binding site in McjC

The AF2 predicted McjA/McjB/McjC ternary structure shows that Glu8, whose side-chain carboxylate will be adenylated to form an active ester, is located next to the ATP binding site in McjC; McjB is omitted for clarity. The overall structure (**a**) and close-up view (**b**) of the McjA/McjC complex are shown. The ATP binding site in McjC (residues 199-204, SGGLDS) is shown in sticks (blue), and Glu8 in McjA is shown in spheres.

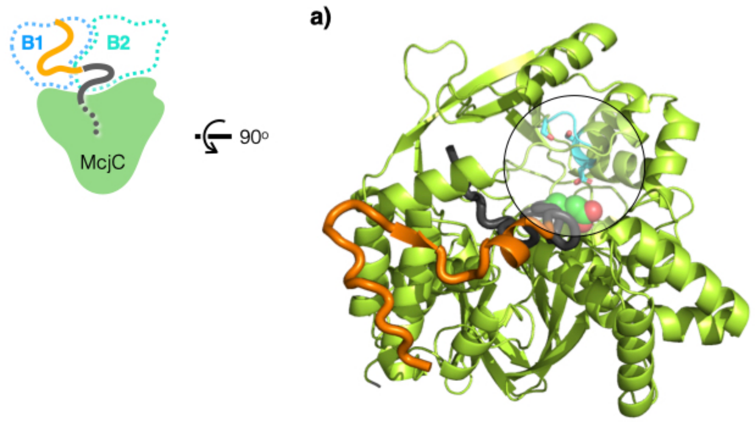

Figure S4 | The workflow for MccJ25 production, QC, and MS confirmation

A typical workflow includes extraction (not shown), inhibition zone assay, HPLC analysis (QC), and confirmation by MS. The results of culturing *E. coli* BL21(DE3) carrying the pTUC202 plasmid (WT) is displayed below to illustrate this process. **a)** The most diluted sample in a two-fold dilution series of the extract to generate a growth inhibition zone with a diameter of 1.0 cm was recorded. **b)** The crude extract was analyzed by HPLC, wherein MccJ25 typically eluted at t_R = 26.3 ± 0.5 min based on our run program. **c)** The peak presumed to be MccJ25 was collected and confirmed by MALDI-TOF MS.

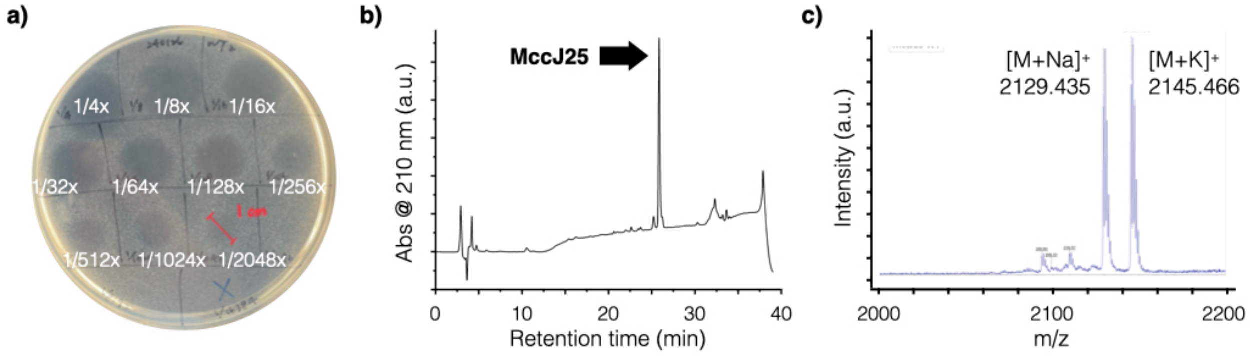

Figure S5 | McjB$_{F67}$ plays an important role in McjB/McjC interaction

The McjB$_{F67}$ residue is involved in at least two types of interactions at the McjB/McjC interface. **a)** Refer to Figure S2 for the hydrogen bond network; shown herein is its cation-π interaction with McjC$_{R438}$. **b)** The guanidinium group to the center of the phenyl ring is approximately 3.5 Å. **c)** The McjB$_{F67R}$ variant produces MccJ25 at a much lower yield compared to the WT.

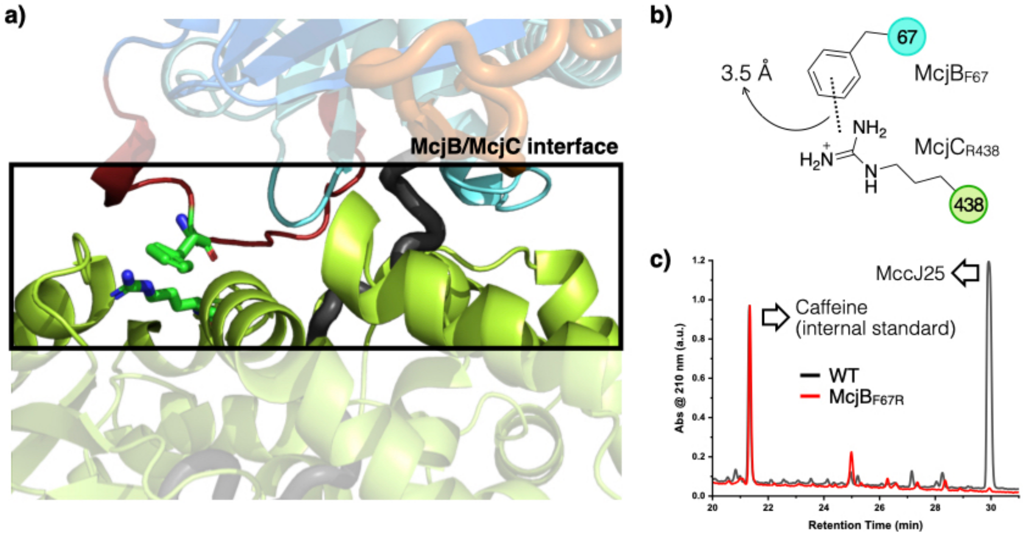

Figure S6 | B enzymes with inverse domain arrangement (sequence alignment)

Four B enzymes with inverse (B2-B1) domain arrangement were identified (see Materials and Methods for details). They belong to lasso peptide BGCs found in the genomes of *Amycolatopsis sulphurea*, *Amycolatopsis panacis*, *Nocardia panacis*, and *Saccharothrix australiensis* (Table S4). The sequences of these four B enzymes were aligned to 10 representative B1 and B2 protein sequences (Table S3). The results clearly showed that all B2 proteins mapped to the N-termini and all B1 proteins mapped to the C-termini of these inverse B enzymes.

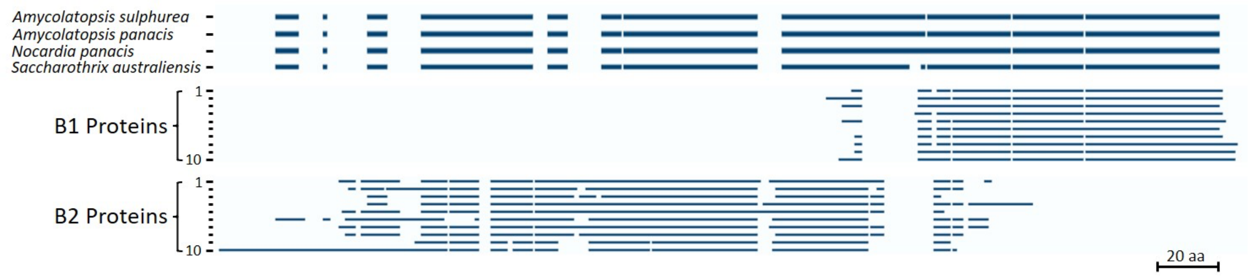

Figure S7 | B enzymes with inverse domain arrangement (AF2 structure predictions)

AF2 predicted structures of candidate inverse B enzymes supported the notion that their domain arrangement is in fact opposite to the vast majority of proteins of this kind. In each predicted structure, The N- and C-termini are shown in gray and black spheres, respectively. TbiB1 (PDB: 5V1V) superposition showed that this archetypical RRE mapped to the C-terminus of these B enzymes.[7] The Cys-His-Glu catalytic triad characteristic of a cysteine protease is shown in sticks and clearly mapped to their N-terminal domains. These B enzymes belong to lasso peptide BGCs found in the genomes of *A. sulphurea* (orange, **a**), *A. panacis* (cyan, **b**), *N. panacis* (pink, **c**), and *S. australiensis* (yellow, **d**) (Table S4).

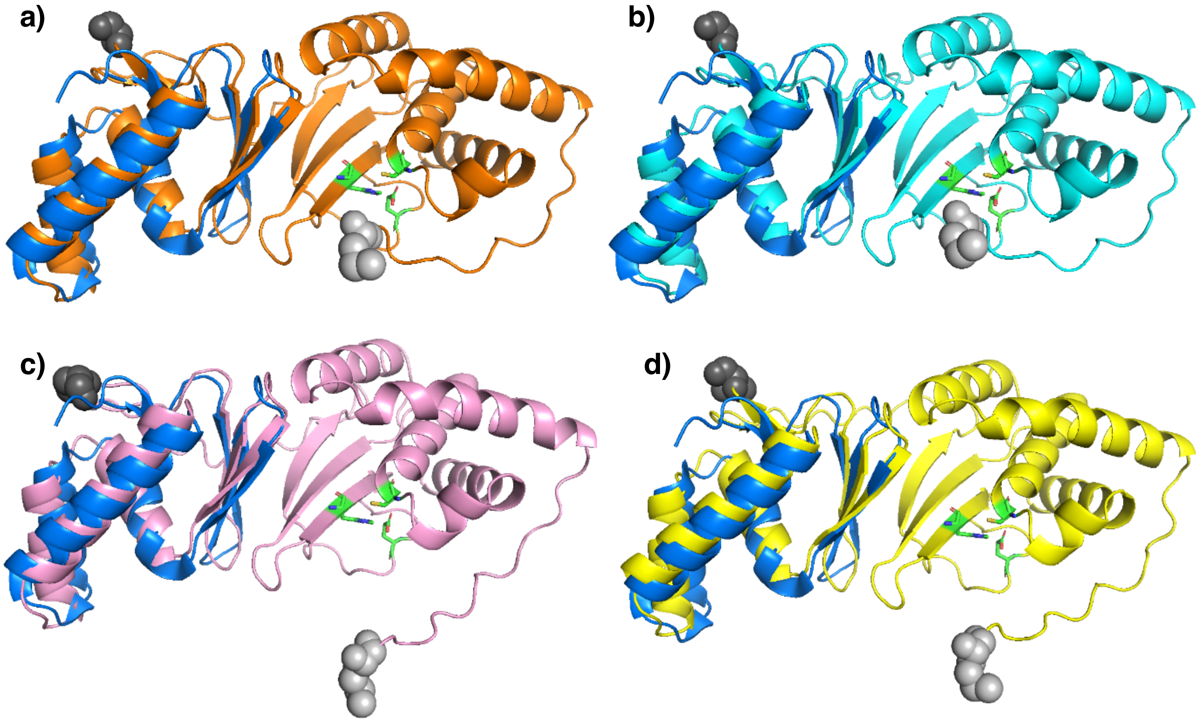

Figure S8 | The McjB $_{S80/81}$ construct is predicted to form a quaternary complex

McjA, McjB$_{1\text{-}80}$, McjB$_{81\text{-}208}$, and McjC were submitted as four separate proteins for AF2 structure prediction. The resulting predicted structure is a quaternary complex that is nearly identical to the McjA/McjB/McjC ternary complex. The leader and core segments of McjA are shown in orange and black, respectively; the first 13 residues of the leader peptide are omitted for clarity. McjB$_{1\text{-}80}$ and McjB$_{81\text{-}208}$ (numbering based on original McjB sequence), which correspond to the RRE and the protease domains, are shown in blue and cyan, respectively. McjC is shown in light green.

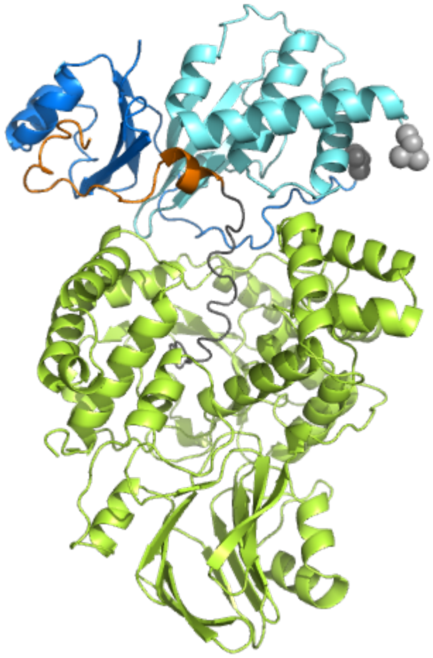

SUPPLEMENTARY TABLEs

Table S1 | Structurally characterized RiPP biosynthetic "B" enzymes

Note that RiPP biosynthetic enzymes containing the RRE are not always designated "B". Nevertheless, throughout this manuscript, we referred to the protein for leader recognition and cleavage as the "B1" and "B2" proteins, respectively.

Name	PDB ID	Microorganism	RiPP Class	Natural Product	Ref.
RRE, non-lasso peptide BGCs					
LynD	4V1T	*Lyngbya aestuarii*	Cyanobactin	Aesturamide	[9]
TruD	4BS9	*Prochloron* sp. 06037A	Cyanobactin	Trunkamide	[10]
ThcOx	5LQ4	*Cyanothece* sp. PCC 7425	Cyanobactin	Cyanothecamide	[11]
MibB	5EHK	*Microbispora* sp. 107891	Lanthipeptide	NAI-107	[12]
NisB	4WD9, 6M7Y	*Lactococcus lactis* subsp. *lactis*	Lanthipeptide	Nisin	[13]
McbB	6GOS, 6GRH	*Escherichia coli*	LAP	Microcin B17	[14]
MccB	3H9G, 6OM4	*Escherichia coli*	Microcin	Microcin C7	[15]
PaaA	5FF5	*Pantoea agglomerans* Eh318	Pantocin	Pantocin A	[16]
PqqD	3G2B, 5SXY	*Xanthomonas campestris* pv. *campestris*	PQQ	Pyrroloquinoline quinone (PQQ)	[17]
CteB	5WGG	*Acetivibrio thermocellus* ATCC 27405	Ranthipeptide	Thermocellin	[18]
SkfB	6EFN	*Bacillus subtilis* subsp. *subtilis* 168	Sactipeptide	Sporulating killing factor (SKF)	[19]
SuiB	5V1T	*Streptococcus suis*	Streptide	Streptide	[20]
TbtB	6EC7, 6EC8	*Thermobispora bispora* DSM 43833	Thiopeptide	Thiomuracin	[21]
Lasso peptide "B1" proteins					
TbiB1	5V1V, 5V1U	*Thermobaculum terrenum* ATCC BAA-798	Lasso peptide	Therbactin α and β	[7]
TfuB1 (FusB1)	6JX3	*Thermobifida fusca*	Lasso peptide	Fusilassin (also known as fuscanodin)	[8]
Lasso peptide "B2" proteins					
None available					

Table S2 | DNA primers used in this study

	**Name**	**Sequence** (5' to 3')
MBP fusion	mcjB_MBP_F1_Gib	GGCGGTAGCAGTAACAATTCATCCATGAAGATTGAGGAAGGTAAACTGGTG
	mcjB_MBP_R1_Gib	GACATTAAATATTTCCATAACTAGGTCTGCGCGTCCTTCA
	mcjB_MBP_F2_Gib	TAGTTATGGAAATATTTAATGTCAAGTTAAATGATACTTC
	mcjB_MBP_R2_Gib	GGATGAATTGTTACTGCTACCGCCTCCTATCTCTGCAATAACAGATAATTTATCCCGC
	mcjC_F1_Gib	TTATGGAAAGAAAACAGAAAAACTCATTA
	mcjC_R1_Gib	ACCTTTATAATCAATGGATTGAAGTTG
	MBP_F1_Gib	CTTCAATCCATTGATTATAAAGGTGGAGGTGGCGGGTCAGGAGGTGGCGGGTCTATGAAG ATTGAGGAAGGTAAACTGGTGAT
	MBP_R1_Gib	GTTTTTCTGTTTTCTTTCCATAATTAGGTCTGCGCGTCCTTCAGCG
Circular permutation	mcjB_1-60_F1_Gib	GCAGAGATAGGCGGTAGCTCTGGCGGTATGATCCGTTACTGCTTAACCAGTTATAGAG
	mcjB_1-60_R1_Gib	TTCCATAACTAAGAAACACTTCCAATATGATATTCACTGTCAAA
	mcjB_61-208_F1_Gib	GTGTGCAAAATGCGTAATAGTGACACTTCTTTTCTTGAAGAAC
	mcjB_61-208_R1_Gib	ACGGATCATACCGCCAGAGCTACCGCCTATCTCTGCAATAACAGATAATTTATCCCGC
	mcjC(61)_F1_Gib	GTTTCTTAGTTATGGAAATATTTAATGTCAAGTTAAATGATACTTCAATTAGA
	mcjC(61)_R1_Gib	ATTACGCATTTTGCACACTCCCTCCTG
	mcjB_1-70_F1_Gib	GCAGAGATAGGCGGTAGCTCTGGCGGTATGATCCGTTACTGCTTAACCAGTTATAG
	mcjB_1-70_R1_Gib	TTCCATAACTATTCTTCAAGAAAAGAAGTGTCACTATTACGAG
	mcjB_71-208_F1_Gib	AGTGTGCAAAATGCGCTGGTTTCTACCAGAACCTG
	mcjB_71-208_R1_Gib	ACGGATCATACCGCCAGAGCTACCGCCTATCTCTGCAATAACAGATAATTTATCCCGC
	mcjC(71)_F1_Gib	GAAGAATAGTTATGGAAATATTTAATGTCAAGTTAAATGATACTTCAATTAG
	mcjC(71)_R1_Gib	CCAGCGCATTTTGCACACTCCCTCCTG
	mcjC(81)_F1_Gib	AAAACATAGTTATGGAAATATTTAATGTCAAGTTAAATGATACTTC
	mcjC(81)_R1_Gib	AGAACACTTATATAACATTTTGCACACTCCCTCCTG
	mcjB_1-80_F1_Gib	GGCGGTAGCTCTGGCGGTATGATCCGTTACTGCTTAACCAG
	mcjB_1-80_R1_Gib	TTTCCATAACTATGTTTTGTCAGGTTCTGGTAGAAAC
	mcjB_81-208_F1_Gib	ATGTTATATAAGTGTTCTCTATTTAAACGATTTATATTATTACTCAAAGTCTT
	mcjB_81-208_R1_Gib	ACCGCCAGAGCTACCGCCTATCTCTGCAATAACAGATAATTTATCCCGCA
Splitting	mcjB2-61_F	AAGGAGATATACCATGCGTAATAGTGACACT
	mcjB1-60_R	CTTAAAGTTAAACTTAAGAAACACTTCCAAT
	mcjB2-71_F	AAGGAGATATACCATGCGCTGGTTTCTACCA
	mcjB1-70_R	CTTAAAGTTAAACTTATTCTTCAAGAAAAGAAGTG
	mcjB2-81_F	AAGGAGATATACCATGTTATATAAGTGTTCTCTATTTAAAC
	mcjB1-80_R	CTTAAAGTTAAACTTATGTTTTGTCAGGTTCT
Synthetic rescue	A_T36M_F1_Gib	GTCCTGCACCACCTTTCATGAGTTGCGA
	A_T36M_R1_Gib	CTTTTTTTTCAATATTCCAGCTATAGTAAAAGACTTTG
	A_T36M_B_M108_F1_Gib	GAATATTGAAAAAAAAGGGATGGCATGGATTTTC
	A_T36M_B_M108_R1_Gib	AAAGGTGGTGCAGGACATGT
	A_T36M_B_M108T_F1_Gib	GAATATTGAAAAAAAAGGGACCGCATGGATTTTCATAAGTAATAAAAAAGAGAA
	mcjA_T36F_F1	CACCACCTTTAAAGAGTTGCGATGCTGATTTTTTTATTTGTATAAC
	mcjA_T36F_R1	CAGGACATGTGCCTGAGTATTTTGTG
	A_T36F_B_F23T_F1	ATAATTAATGATAGTACCAGCATAGTGCCTGACGCAG
	A_T36F_B_F23T_R1	ATCCAGGATAACAAGATCCTCTCTATAAC

	Name	Sequence (5′ to 3′)
McjA/McjC recognition	mcjC_K337E_F	GAATTTTCCAATCTCGAGGGAAAGAGATATAAAG
	mcjC_V331_R	TACTATTTTTTTATGCATAAACATCAAGCCATGAC
	mcjC_S440Y_F2	CAAACGGTCATATTCACAGCTAATATTC
	mcjC_K435_R2	GTTTTTTTCCAGAAAATATCTGAACC
McjB/McjC interface	mcjB_F67R_F2	GACACTTCTCGTCTTGAAGAACGCTGGTTTCTAC
	mcjB_N62_R2	ACTATTACGAGAAACACTTCCAATATGATATTCAC

Table S3 | Representative B proteins for McjB sequence alignment (Figure 4)

Throughout this manuscript, we referred to the protein for leader recognition and cleavage as the B1 and B2 proteins, whereas in some literature they are named the E and B proteins, respectively.

Microorganism		Accession no. [ab]	Sequence	Length
Francisella cf. *tularensis* subsp. *novicida* 3523	B1	WP_014548150.1 (YP_005823897.1)	MSKINLKDKITRNNDFYSSEIDNELIMMDISSNNFYTTGEIGN<br>RIWELLEFETSCENICQQLCQEYDVSIEQCQQDTLSFLNQLLE<br>NKAIQLKK	94
	B2	WP_014548149.1 (YP_005823896.1)	MIIQKVYRKLRTATKMPLKKKVWFIILYPISGIGRFASLVLSL<br>KKIFWYLGYTHGNAQLCIPASEEQLILAYKISKTATLVSKYVP<br>WESKCLIDAIMVKTLLKYYKIPYVIHIGMIKTNEENKPFMGHA<br>WVKVADQIVIGGDGQGCKNYSINCTITSINFKTKA	164
Legionella pneumophila subsp. *pneumophila* ATCC 43290	B1	WP_010948633.1 (YP_005187271.1)	MSNHLQQHKYQFEYKTNTILERYDLVFFSSVDEDLILLDEEAS<br>HYLVVNSVGYKIWNLLESKKTVSQLVSGLMEIYKVDFDTCFND<br>IKAFIQQMVQHKLIKVCS	104
	B2	WP_014327030.1 (YP_005187270.1)	MTEIKKIRYAHLFLVLLDKGKTRVHVAVLIVYYLIVSRIITWL<br>YPFNRYAKSFGHKHAETLYEDFDHLYWLKCCQKLFPKINRLLP<br>WKSACLQQSVAAFLLLRRKNMPTTIYFGMKREGDEVIGHVWLR<br>CGKHYITGGNGVGYTIFCTYALDRVKN	156
Chitinophaga pinensis DSM 2588	B1	WP_012792509.1 (YP_003124542.1)	MSESVQTSLCKETIVRRNERNFLVSRIGEEVVLMDIHNGQYIG<br>LNAVGSAIWEKLEQPVAIHDVVNALMQEYAISMQLCEQETLLF<br>LQKMMQHRMLIVD	99
	B2	WP_012792507.1 (YP_003124540.1)	MKMKRIFKASFLFAEAWVYLGVARVMLVFMPFRKIAPMLGETI<br>YTAPAALPSSLRPHRIRAAIRRAASCAPWRTKCFEQALAGKLM<br>LRYRRMSGVIFFGVNKTGAELKAHAWLESEGVIITGAKGIEQY<br>TVIARFKN	137
Citromicrobium sp. JLT1363	B1	ZP_08702801.1	MAIESDCTIRKSENFVETVVDEELVLMHIVDGRFYALKDTGRH<br>AWNLLDEHARFGDLVEAVRGDYDVSEATCREELGKLFDDLRER<br>TLVSIDC	93
	B2	ZP_08702804.1	MRIVRFLRRKLGEAEIWVSLAVAWVLVRFVPFNWWRKSIGPIG<br>GEGMAALRVDEAATRRAYGLGRAVERIADRAWFAPVCLPRAIA<br>ARWMLNRRGIPSRIVFGSRRNDDPAGRKLLFHAWLKVADVVVT<br>GAQGHHAFVPFEKRSTENSSDNSPGEDRPERELSDAIATRKF	171
Citromicrobium sp. JLT1363	B1	ZP_08702967.1	MPKASEITLATTLGKAEQVLSSSVDDGLLLMDMDTGDYHHFDD<br>IASSIWSRIDGDKSVGDVCLELQDEFQVDPAQCEEDTLEFLRQ<br>AHRANLVADQSA	98
	B2	ZP_08702964.1	MTGYIGRKLTVFWKLSVPAKLLLLPAWLSLCVAAGIFRFVRFG<br>RLGQLLGRNIGAGACCPLASPAAVDRAIAISSAVQIASRYTPF<br>RSDCVPQAIAGQFLCRALNVPSALHFGVGNRVASAVSGESMQA<br>HAWLVVGPIFVTGGRSFKTHTTTACFVAR	158
Erythrobacter sp. NAP1	B1	ZP_01040577.1	MQLSDRFAVSADVVAREVGGEMVLLDLSSGQYFGLDPVGGRIW<br>ELLAEGPRELAELCDSVEAEFEAPRDRIEADLLALAKQLKDQD<br>LIVAA	91
	B2	ZP_01040581.1	METAQSSPVPAKGQKPTLADIGWLASYSARGLGELFRARLIFR<br>NFKASDIPLRNRAAANAGRPTSASGQPVEAATLARIRYVIPRL<br>SDRLPWRSDCLIQAIAAQNWLCSLGVQSEIQIGVEKPEDGEFG<br>AHAWLMSGGEIITGGDIAQYDVILSESRLKGDSGTKD	166
Gallionella capsiferriformans ES-2	B1	WP_013292802.1 (YP_003846625.1)	MIVELSSIISQGTDPICTEVDGETVMMSIEKGNYYGMNGVGSR<br>IWQLIAEPMSVSVLCGILLDEFCIDKKTCEVDALIFLNKLEEQ<br>NLIQVRS	93
	B2	WP_013292807.1 (YP_003846630.1)	MIFSTLLRKVRNFAHLSWFVKTWFLPVWFLLGVSRSLVLIIPF<br>RHLAPRLGVRTGILPWVALVDSGREARALSIARVVQTAASYTP<br>WVSNCFPQAVVARILLGIYCVPYCLFFGVTRDPTGSTLKAHAW<br>VTAGRVRVTGGESFGQFTVLGCFVSPCLAHAISQIDQ	166

Geotalea uraniireducens Rf4	B1	WP_011938275.1 (YP_001230131.1)	MSIDMESLIVRNNEMMSSAMDRELVILNMAKGNYIGLDEIGRR<br>IWELLETPLGAEELCGLLGREFDASPEQITADVLPFLVELESE<br>GMVHDVTGGRSA	98
	B2	WP_011938274.1 (YP_001230130.1)	MMLRADDLRKLFSLTLAEVCLLLEAAFWLGICRLAIPLLPFRW<br>IAPYLGTHMAESASVLDPHGREVPLAVSRAIVRAAWRLPWDCK<br>CLAQAMTGKAMLKRRGVPSTLYLGVAKDKEQLAAHAWLRCGDI<br>ILTGGQGKERFTVVSSFGDDIRSRG	154
Mesorhizobium amorphae CCNWGS0123	B1	ZP_09090635.1	MNWNPSDRDCVSATNDAVACEFGNGLALLNLKSNIYYSLNSVG<br>AYIWDLIQEPRPIADIRSAVLTRYDVDPERCKADVDGLLKGLA<br>EAGLARLHHEELV	99
	B2	ZP_09090636.1	MLFLAHCLLVVAAVRLGLTLFSYNRLRRRVTQLDAPHEAGIGD<br>LRRVAWGVAAAARLVPYASCLTQAISGQYILARQGNGSKIRIG<br>IERDTGTQLKAHAWLISGNHIVLGGSINGFAHLVDHGQ	124
Mesorhizobium ciceri bv. *biserrulae* WSM1271	B1	WP_013528114.1 (YP_004139466.1)	MNGELSDRDLGDHDSVCATKDAVACEFGDGLALLNLKSNVYYS<br>LNGVGAFIWDLIQEPRSIGDIRSAVLARYNVDPARCQADVDAL<br>LKGLADNGLARRHHEALV	104
	B2	WP_013528115.1 (YP_004139467.1)	MRHLSKPVQHKSLVSRALVSRALSSRATALRQEPARETNVKQT<br>KRGGSRRGLVRIFSLSGSEMIFLGYCLLVVATVRLGLTLLSYN<br>RVRSLVTRLDAPQCASMGELRRVAWGVAAAARFVPYATCLTQA<br>LSGQYILARQGNESTIRIGIERGTGEQLKAHAWLVSDNHIVLG<br>GSIDGFAHLVDHGSR	187
Novosphingobium aromaticivorans DSM 12444	B1	WP_011446880.1 (YP_498512.1)	MSLVLRKVDSSFCATEVDGELVMIHSGTGKFFSLKGVGLEIWN<br>ALDRQANLEAISADLVQEYGISAEECAAAVEGFASQLVAAGFA<br>EFV	89
	B2	WP_011446879.1 (YP_498511.1)	MSDQQDGRLDSSGGARIWWLRFNVLKATLALTAARLVVSCLPL<br>RTWSPVLGAMSGSPVATQIPESPPAEAFDGPRHAHARLLAGCV<br>ERAAERLPGTSKCLPKAVALQVLLRLAWIPSRLVIAFHVTDRT<br>GPDAYHAWTEVCGEMLVGECNRAEYRSIMTFDQPRPVRDRT	170
Psychromonas ingrahamii 37	B1	WP_011770931.1 (YP_943973.1)	MNNNILTLNSVISRAENVLDCTVDNEMVLMSITSGDYINLNAQ<br>ASAIWSVISEAQPVFIIVEKLLAQFEVELEDCQQQTLAFLNTL<br>KQESLIKIT	95
	B2	WP_011770934.1 (YP_943976.1)	MFSIKNKFSTFCTLSSRQKCWFLILLLYSGLLRFCLLFLPFHL<br>LSSILGKQFSNLQCCSVIKDNQLHYLPEIGQITRLVERYSPWE<br>SKCLIQAMLAASLYRYYQIPYVIYLGVLKQNDSKELLKAHAWS<br>LVGDKIITGAKGHKKFTIINTYISPLINH	158
Rhodobacter sphaeroides 2.4.1	B1	WP_011337564.1 (YP_352614.1)	MSIRAAEGCVPCAFGDGIAIFDTSSNSYFSMNAVGEFVWSQLD<br>QPITLEDLVGRVADRYRIEPTVCEGDIRKLVDDLAAHRLVTLS	86
	B2	WP_011337565.1 (YP_352615.1)	MRRLRRILSLRPAEAWALCQALVTVAAVRLAIARRRTDEVRAA<br>TAALGAERQAPQSDLRVVAWSVTAAARLIPGATCLTQALAGQR<br>ILARKGYASTVRLSLPAGRDSDFRPHAWLLAGNVIALGGTATD<br>YRHHRALLDYESSGRADPVSEPATGAGQ	157
Rhodospirillum rubrum ATCC 11170	B1	WP_011390773.1 (YP_428107.1)	MTTPEPLTPGSLVGWHPEQVTAAVDGEVIVMGLIRGQYVGLDD<br>IGSVLWTLLEQPRTVRQLCDDLGRRYQGDPATMSADVVAFLED<br>LRALDLIEVLDAAPLSDP	104
	B2	WP_011390771.1 (YP_428105.1)	MTARRLRRGLSLPAAERWLAVEGLAMLAVSRAVLALLPFRIAM<br>RWLGLRLDRGTGRMGGEAEETPATPSVLLVGAAVRRAAAIAPF<br>RAVCLQQAVAAALMLRRRGHAAQVHFGVTRDQDGNIIAHAWTR<br>CQGEVVTGEQQMSQYQPISVFVT	152
Rhodospirillum rubrum ATCC 11170	B1	WP_011388578.1 (YP_425911.1)	MVTIDTVLVRTDHCLVSEIDNELVMMDVQSGHYFTLDAIGNNI<br>WNRLEQPVRVGDLCVILEQAYAAPLDVITADVLRLLDSMAGEG<br>LVKVAA	92
	B2	WP_011388577.1 (YP_425910.1)	MNRAGSLRRLRSRLFWLEAVLALCIAWLLVFRLPFRLLARLFG<br>GIADPGPALAGSDPQPAPVLARARGVGQGVDAMSVRLPWHSTC<br>LVRCVAGRMMLTRRGIAGHILFGVDRRGGTLTAHAWLVTGGEP<br>VIGGAEATGFTPNACLYARPRIL	152

Roseobacter denitrificans OCh 114	B1	WP_011569924.1 (YP_683999.1)	MVSENCYVAQSDVVDCDIGGDRALLHLQTNTYFTMNATASALW<br>LGLSEPKSLNEMVQIVTEKFDVTDDQCRADIETLVGQMVEANV<br>VKVVADEAK	95
	B2	WP_011569923.1 (YP_683998.1)	MRAIRWAGIYTLSLLVVLVVRVGLWVTRYQRIRAALVRPCPDD<br>PQLERRATVARVTHAVARIARFIPDASCLTQTISCQAILSWKG<br>IPSTITMGLKKEDETTLKAHAWLTWNGQVVLEGNEGTLLDFNK<br>ILDLPTPVRSSVSL	143
Shewanella sp. HN-41	B1	ZP_08567200.1	MMDVSQGKYFGLNSFATQIWSLLASPKSIDDLVVELMSLFDVT<br>EDVCRADTTLFLQQLVDKQLVKVTTAA	70
	B2	ZP_08567205.1	MQQMKTVIKKLKAFASREPFYQVRFVPTWLLLGFARLTVLLLP<br>FARLAPYLGVSVASENAQHNIPNLSVALTTSDMQRAKQISRLV<br>VNTARYCPWKANCFAQAIVARIWLGWYGLPYRLYFGLRRDDSS<br>LKAHAWVCCSTIFVSGGNGFIHHSVVGCYGSKCDARIEANG	170
Sphingobium yanoikuyae ATCC 51230	B1	EKU73220.1	MTIAWPSATIVAADPNIVFCDLDNERVLLDLTSSRYFALNRVG<br>VHVWETIATPTSVGALQDNIEQRFDVRPEQCRKDLESLLSNLS<br>QFTLISVQHAPAS	99
	B2	EKU73219.1	MRRRPDRTLAGLLLALLLPVALPVVLAVRLALSLTSYARLCEW<br>LPQSTGQRNPLWVRQVARAVSIVSRLVPGATCLTQAVATRTLL<br>AWMGHESWIRIGVRRSEDGSFQAHAWLLDHRARPIIGGRRQDL<br>AGFNVLADLDGLVRQ	144
Sulfurovum sp. NBC37-1	B2	WP_012083841.1 (YP_001359374.1)	MDLNKKVKFADMVFAQEVDGEMVLLDMNSENYFGLDAVGTDIW<br>VAMQQKKNLQEVLESLLEQYDVEEEVIKQDLEAFVHKLVESGL<br>VEVVA	91
	B2	WP_012083842.1 (YP_001359375.1)	MIRKFKKFTQLPSEEKKLFIEACVTLGKIRAAILTVSFKRLTC<br>SLEHKTTVEKLTPLSEEEVHTARLVGQAIVRASAYTPWESACL<br>TQSLTAQKMLQKRGISGVFYLGVAKDEENEAKMKAHAWAQCGD<br>AIVTGGRGHEAFTVLSVFGW	149
Xanthomonas axonopodis pv. *punicae* str. LMG 859	B2	ZP_10261506.1	MRRRDPLDHLPPHVPSAMLSSSSALQSRKQAPQPRLLARTAID<br>AETAVLRQRSAARADRWQ	61
	B2	ZP_10261507.1	MSMPRLLVRGCRMLAAVGRLPRFERLLLVPAWVLLGLVRAAVR<br>WLGFRRLAPWLGDQVSVPPALPGLDARAERRALQIGRTVRLAA<br>RYTYWDSNCLAQALAAHYLLGLFRVAHLLCLGVTRSDDGAQLQ<br>AHAWVLAGSARVTGGRASERFTRVGCFVPRARQAR	164

[a] This collection of proteins was reported in the Supporting Information of the publication by Marahiel and co-workers;[6] [b] A number of proteins have been assigned new accession numbers and the originals were given in parentheses.

Table S4 | List of B enzymes with inverse domain arrangement

Microorganism	Accession no.	Sequence	Length
Amycolatopsis sulphurea	WP_098513787.1	MSTPFAAPERVRLPLRTRLSSLMAIAAATPLTRLPPRLLRPILELA<br>SRRARPASAAQTSAAREAIVASSIRCAVNGCLKRSIATALLCRTRG<br>VWPTWQLGARTMPFGAHAWVEAEGTMIGEPVPEGYYVPLITVAPPR<br>PEAPRGRWRTTGGTGILRLHRDALTADTGDGAVLLNQRTGHYWQLN<br>HTGADSLRRLLAGQSVQDAAHDYAAEYGIEPSEAHQDITAMTGQLL<br>EAGFLTRA	238
Amycolatopsis panacis	WP_120026939.1	MSTPFAAPERVRLPLRTRLSSLMAIAAATPLTRLPPRLLRPILELA<br>SRRARPASAAQTSAAREAIVASSIRCAVNGCLKRSIATALLCRTRG<br>VWPTWQLGARTMPFGAHAWVEAEGTMIGEPVPEGYYVPLITVAPPR<br>PGASRGRWRTTGGTGILRLHRDALTADTGDGAVLLNQRTGHYWQLN<br>HTGADSLRRLLAGQSVQDAAHDYAAEYGIEPSEAHQDITAMTGQLL<br>EAGFLTRA	238
Nocardia panacis	WP_120042767.1	MSTPCAPPERIRLPWRQRVLPLIAVAVAAPLTILPPKNLRWVLEFA<br>RRGARPAPADQTSRARQAIMAVSLRCAVNGCLQRSIATALLCRVRG<br>VWPTWRLGVCTAPFGAHAWVEAEGRMIDEPMPAGHYTPMLTVAPAQ<br>VAADPDHYSEMGTGTGDFRLHRQVLSVGTSDGAVLLHLRTGHYWQL<br>NPVGLDSLRHLLSGRSVEDIATDFAAEYAIDPAGPRRDITVMTDQL<br>REAGFLVGP	239
Saccharothrix australiensis	WP_121006347.1	MSTPYAPPERVRLAPRTRLSALAAVAVAAPLTRLPPKVLRAVLALA<br>SRGARAATAAQASAAREAVVASSLRCAVNGCLQRSIATALLCRARG<br>TWPTWRLGARTTPFGAHAWVEAEGRMIDEPLPDGYYVPLITVGPPT<br>PGSTCRERTAGILRLHHDALSAETDDGAVLLNQRTGDYWQLNHTGV<br>DTLRRLLSGQTPDDAAEHYAAHYDIDPALAHRDILAMTDRLLDAGF<br>LTRT	234

REFERENCEs

[1] a) J. O. Solbiati, M. Ciaccio, R. N. Farias, R. A. Salomon, Genetic analysis of plasmid determinants for microcin J25 production and immunity. *J. Bacteriol.* **1996**, *178*, 3661-3663; b) J. O. Solbiati, M. Ciaccio, R. N. Farias, J. E. Gonzalez-Pastor, F. Moreno, R. A. Salomon, Sequence analysis of the four plasmid genes required to produce the circular peptide antibiotic microcin J25. *J. Bacteriol.* **1999**, *181*, 2659-2662.

[2] a) J. Jumper, et al., Highly accurate protein structure prediction with AlphaFold. *Nature* **2021**, *596*, 583-589; b) AlphaFold2.ipynb - Colaboratory https://colab.research.google.com/github/sokrypton/ColabFold/blob/main/AlphaFold2.ipynb#scrollTo=ADDuaolKmjGW (accessed March 13, 2024).

[3] E. Krissinel, K. Henrick, Inference of macromolecular assemblies from crystalline state. *J. Mol. Biol.* **2007**, *372*, 774-797.

[4] P. H. Chen, L. K. Sung, J. D. Hegemann, J. Chu, Disrupting transcription and folate biosynthesis leads to synergistic suppression of *Escherichia coli* growth. *ChemMedChem* **2022**, *17*, e202200075.

[5] A. M. Kretsch, M. G. Gadgil, A. J. DiCaprio, S. E. Barrett, B. L. Kille, Y. Si, L. Zhu, D. A. Mitchell, Peptidase activation by a leader peptide-bound ripp recognition element. *Biochemistry* **2023**, *62*, 956-967.

[6] J. D. Hegemann, M. Zimmermann, S. Zhu, D. Klug, M. A. Marahiel, Lasso peptides from Proteobacteria: Genome mining employing heterologous expression and mass spectrometry. *Biopolymers* **2013**, *100*, 527-542.

[7] J. R. Chekan, C. Ongpipattanakul, S. K. Nair, Steric complementarity directs sequence promiscuous leader binding in RiPP biosynthesis. *Proc. Natl. Acad. Sci.* **2019**, *116*, 24049-24055.

[8] a) T. Sumida, S. Dubiley, B. Wilcox, K. Severinov, S. Tagami, Structural basis of leader peptide recognition in lasso peptide biosynthesis pathway. *ACS Chem. Biol.* **2019**, *14*, 1619-1627; b) A. J. DiCaprio, A. Firouzbakht, G. A. Hudson, D. A. Mitchell, Enzymatic reconstitution and biosynthetic investigation of the lasso peptide fusilassin. *J. Am. Chem. Soc.* **2019**, *141*, 290-297.

[9] J. Koehnke, et al., Structural analysis of leader peptide binding enables leader-free cyanobactin processing. *Nat. Chem. Biol.* **2015**, *11*, 558-563.

[10] J. Koehnke, et al., The cyanobactin heterocyclase enzyme: a processive adenylase that operates with a defined order of reaction. *Angew Chem Int Ed Engl* **2013**, *52*, 13991-13996.

[11] A. F. Bent, G. Mann, W. E. Houssen, V. Mykhaylyk, R. Duman, L. Thomas, M. Jaspars, A. Wagner, J. H. Naismith, Structure of the cyanobactin oxidase ThcOx from Cyanothece sp. PCC 7425, the first structure to be solved at Diamond Light Source beamline I23 by means of S-SAD. *Acta Crystallogr. D Struct. Biol.* **2016**, *72*, 1174-1180.

[12] M. A. Ortega, Y. Hao, M. C. Walker, S. Donadio, M. Sosio, S. K. Nair, W. A. van der Donk, Structure and tRNA specificity of MibB, a lantibiotic dehydratase from Actinobacteria involved in NAI-107 biosynthesis. *Cell Chem. Biol.* **2016**, *23*, 370-380.

[13] M. A. Ortega, Y. Hao, Q. Zhang, M. C. Walker, W. A. van der Donk, S. K. Nair, Structure and mechanism of the tRNA-dependent lantibiotic dehydratase NisB. *Nature* **2015**, *517*, 509-512.

[14] D. Ghilarov, C. E. M. Stevenson, D. Y. Travin, J. Piskunova, M. Serebryakova, A. Maxwell, D. M. Lawson, K. Severinov, Architecture of microcin B17 synthetase: An octameric protein complex converting a ribosomally synthesized peptide into a DNA gyrase poison. *Mol. Cell.* **2019**, *73*, 749-762 e745.

[15] C. A. Regni, R. F. Roush, D. J. Miller, A. Nourse, C. T. Walsh, B. A. Schulman, How the MccB bacterial ancestor of ubiquitin E1 initiates biosynthesis of the microcin C7 antibiotic. *EMBO J* **2009**, *28*, 1953-1964.

[16] S. V. Ghodge, K. A. Biernat, S. J. Bassett, M. R. Redinbo, A. A. Bowers, Post-translational Claisen condensation and decarboxylation en route to the bicyclic core of pantocin A. *J. Am. Chem. Soc.* **2016**, *138*, 5487-5490.

[17] T. Y. Tsai, C. Y. Yang, H. L. Shih, A. H. Wang, S. H. Chou, Xanthomonas campestris PqqD in the pyrroloquinoline quinone biosynthesis operon adopts a novel saddle-like fold that possibly serves as a PQQ carrier. *Proteins* **2009**, *76*, 1042-1048.

[18] T. L. Grove, P. M. Himes, S. Hwang, H. Yumerefendi, J. B. Bonanno, B. Kuhlman, S. C. Almo, A. A. Bowers, Structural insights into thioether bond formation in the biosynthesis of sactipeptides. *J. Am. Chem. Soc.* **2017**, *139*, 11734-11744.

[19] T. A. J. Grell, W. M. Kincannon, N. A. Bruender, E. J. Blaesi, C. Krebs, V. Bandarian, C. L. Drennan, Structural and spectroscopic analyses of the sporulation killing factor biosynthetic enzyme SkfB, a bacterial AdoMet radical sactisynthase. *J. Biol. Chem.* **2018**, *293*, 17349-17361.

[20] K. M. Davis, K. R. Schramma, W. A. Hansen, J. P. Bacik, S. D. Khare, M. R. Seyedsayamdost, N. Ando, Structures of the peptide-modifying radical SAM enzyme SuiB elucidate the basis of substrate recognition. *Proc. Natl. Acad. Sci.* **2017**, *114*, 10420-10425.
[21] I. R. Bothwell, D. P. Cogan, T. Kim, C. J. Reinhardt, W. A. van der Donk, S. K. Nair, Characterization of glutamyl-tRNA-dependent dehydratases using nonreactive substrate mimics. *Proc. Natl. Acad. Sci.* **2019**, *116*, 17245-17250.
